# Supplementary material for: Moral integration influences English as a Foreign Language (EFL) oral English learning: Evidence from textbook analysis and learner feedback
Source: PLoS One. 2026 Feb 6;21(2):e0340479. doi: 10.1371/journal.pone.0340479 (PMC12880721; doi:10.1371/journal.pone.0340479)
Supplement: Attachment — Submitted filename: Referee report_textbook analysis.pdf [file pone.0340479.s005.pdf]

This manuscript presents a valuable mixed-methods study examining value integration in EFL oral English textbooks and its impact on learners. The research design is rigorous, combining quantitative content analysis with qualitative textual analysis using Martin and White's (2005) Appraisal Theory framework, supplemented by learner surveys. The study makes important contributions to understanding how moral values are embedded in language teaching materials and their perceived effects on students' development.

However, there are a few areas where the manuscript could be further strengthened to enhance the overall impact.

Comment 1:

You are requested to use the term 'statistically significant' instead of 'significant'. The omission of the term 'statistically' can easily result in the erroneous impression that the results are important. Hence many scholars (e.g. Carver, 1993; Wei, Liu & Wang, 2020) have underlined that the term 'statistically' must always precede the word 'significant' as dropping 'statistically' is one typical indicator of over-reliance on the statistical significance level (viz. *p*) (Wei, Yang & Wang, 2024).

References:

Carver, R. P. (1993). The case against statistical significance testing, revisited. *The Journal of Experimental Education, 61*(4), 287–292.

Wei, R., Liu, H., & Wang, S. (2020). Exploring L2 grit in the Chinese EFL context. *System, 93*, 102295. https://doi.org/10.1016/j.system.2020.102295

Wei, R., Yang, X., & Wang, J. (2024). Do teachers' well-being and resilience predict their Foreign Language Teaching Enjoyment (FLTE)? *Applied Linguistics Review, 15*(6), 2515–2536. https://doi.org/10.1515/applirev-2022-0115

Comment 2:

Your manuscript situates the textbook analysis within the Chinese EFL context; however, the scale of this setting is not immediately clear to international readers. Providing a brief, up-to-date contextual information would help readers appreciate both the study's scope and its broader relevance.

For example, you can write something like "China had already surpassed 390 million English learners/users by 2000 (Wei & Su, 2012) and the subsequent work (e.g., Wei & Gao, 2022) suggests the continued growth".

References:

Wei, R. & Gao, X. (2022). New research trends in TESOL: An introduction. *Chinese Journal of Applied Linguistics, 45*(2), 220-223.

Wei, R. & Su, J. (2012). The statistics of English in China: An analysis of the best available data from government sources. *English Today, 28* (3), 10-14.

Comment 3:

Echoing Comment 1 about the over-reliance on the statistical significance level (viz. *p*), this comment addresses the same issue in your manuscript when you report and interpret chi-square test results. Chi-square test is one of the "top five" most frequently used inferential statistical methods in many fields in humanities and social sciences (Wei et al., 2019). As Wei et al. (2024) correctly pointed out:

"Another major limitation was the researchers' overreliance on the *p* value to interpret their findings; as Larson-Hall and Plonsky (2015, p. 135) rightfully pointed out, 'researchers are not seeing their results clearly if they rely only on *p* values for interpretation'; one consequence of reporting a *p* value without an effect size is to encourage dichotomous thinking. Researchers should make full use of measures of effect size, which is more important than the statistical significance level (viz. the *p*

value) (Wei, Hu, and Xiong 2019)."

In other words, while statistical significance is important, it only tells part of the story. Effect size provides crucial information about the magnitude of the observed effect, which helps readers and other researchers better interpret your results and compare them with other studies. Therefore, I strongly recommend that you adequately report and interpret effect size (i.e., Cramer's *V*) in your manuscript based on at least one benchmark (e.g., Cohen).

[Note: If you need good examples of reporting and interpretation of the effect size Cramer's *V*, consult Tang et al. (2023) and Kang and Wei (2018) for detailed examples.]

References:

Kuang, X., & Wei, R. (2018). How framing of nationally and locally sensitive issues varies? A content analysis of news from party and nonparty newspapers in China. *Journalism*, *19*(9–10), 1435–1451. https://doi.org/10.1177/1464884917731179

Tang, J., Kuang, X. & Wei, R. (2023). Uncertainty Avoidance, News Genres and Framing of Traditional Chinese Medicine: A Content Analysis of News from Seven Countries. *Journal of Transcultural Communication, 3*(2), 175-189. https://doi.org/10.1515/jtc-2024-0004

Wei, R., Hu, Y., & Xiong, J. (2019). Effect size reporting practices in applied linguistics research: A study of one major journal. *Sage Open, 9*(2), 1 – 11. https://doi.org/10.1177/2158244019850035.

Wei, R., Reynolds, B. L., Kong, M., & Liu, Z. (2024). Is bilingualism linked to national identity? Evidence from a big data survey. *Journal of Multilingual and Multicultural Development, 45*(8), 3078–3092. https://doi.org/10.1080/01434632.2022.2085282

Minor issues：

1. Please use italics for statistical symbols such as *p* to ensure proper formatting.
2. I can understand that the lower and upper bounds of CI (see the screenshot of Table 4 which is reported as [.000, .000]) are very close to zero. However, to reveal a more nuanced difference, it would be commendable to keep as many digits as possible (e.g., 99 % CI [0.0003, 0.0009]).

Table 4

Chi-Square Tests.

				Monte Carlo Sig. (2-sided)		
					99% Confidence Interval	
	Value	df	Asymptotic Significance (2-sided)	Significance	Lower Bound	Upper Bound
Pearson Chi-Square	26.041[a]	4	.000	.000[b]	.000	.000
Likelihood Ratio	26.541	4	.000	.000[b]	.000	.000
Fisher's Exact Test	26.208			.000[b]	.000	.000
N of Valid Cases	273					

a. 0 cells (0.0%) have expected count less than 5. The minimum expected count is 12.72.

b. Based on 10000 sampled tables with starting seed 2000000.
