# Supplementary material for: Moral integration influences English as a Foreign Language (EFL) oral English learning: Evidence from textbook analysis and learner feedback
Source: PLoS One. 2026 Feb 6;21(2):e0340479. doi: 10.1371/journal.pone.0340479 (PMC12880721; doi:10.1371/journal.pone.0340479)
Supplement: Attachment — Submitted filename: pone-d-25-15954_reviewer.docx [file pone.0340479.s003.docx]

PLOS ONE

# How Does Value Integration Impact EFL Oral English Learning? Textbook Analysis and Learner Perspective

--Manuscript Draft--

| **Manuscript Number:** | PONE-D-25-15954 |
| --- | --- |
| **Article Type:** | Research Article |
| **Full Title:** | How Does Value Integration Impact EFL Oral English Learning? Textbook Analysis and Learner Perspective |
| **Short Title:** | Value Integration in EFL Oral Learning: Textbook & Learner Insights |
| **Corresponding Author:** | Xixi Yang, M.D.  Jiangxi Agricultural University Nanchang, CHINA |
| **Keywords:** | integration of morals and values; textbook analysis; oral English learning; value- driven language instruction; learner outcome |
| **Abstract:** | The research is motivated by the importance of understanding how value education is intertwined with language learning, and how this synthesis contributes to the holistic development of learners. The research employed a mixed-methods approach, which involved categorizing and analyzing verbal texts from the selected EFL oral English textbook series at the college level. According to frequency statistics, the sample textbooks include 273 verbal texts embedded with morals and values, and a Chi- Square Test shows a significant difference of dimension emphasis between different textbook volumes. Within the theoretical framework of Martin and White's (2005) Language Appraisal Theory, a qualitative textual analysis reveals the value significance of lexical-grammatical items as a prominent feature across various teaching modules. Additionally, a questionnaire survey was conducted with EFL learners who used the textbook series for an academic year. The survey showed that values-based language instruction positively impacted morality and personal growth. |
| **Order of Authors:** | Xixi Yang, M.D. |
| **Additional Information:** |  |
| **Question** | **Response** |
| **Financial Disclosure**  Enter a financial disclosure statement that describes the sources of funding for the work included in this submission. Review the [submission guidelines](http://journals.plos.org/plosone/s/submission-guidelines#loc-financial-disclosure-statement) for detailed requirements. View published research articles from [*PLOS ONE*](https://journals.plos.org/plosone/) for specific examples.  This statement is required for submission and **will appear in the published article** if the submission is accepted. Please make sure it is accurate. | Yes |

| **Funded studies**  Enter a statement with the following details:   - Initials of the authors who received each award - Grant numbers awarded to each author - The full name of each funder - URL of each funder website - Did the sponsors or funders play any role in the study design, data collection and analysis, decision to publish, or preparation of the manuscript? |  |
| --- | --- |
| Did you receive funding for this work? |  |
| Please add funding details.  as follow-up to "**Financial Disclosure**  Enter a financial disclosure statement that describes the sources of funding for the work included in this submission. Review the [submission guidelines](http://journals.plos.org/plosone/s/submission-guidelines#loc-financial-disclosure-statement) for detailed requirements. View published research articles from [*PLOS ONE*](https://journals.plos.org/plosone/) for specific examples.  This statement is required for submission and **will appear in the published article** if the submission is accepted. Please make sure it is accurate. | This paper is supported by the Educational Reform Research Project of Jiangxi Agricultural University, with the project title ''Deep Reform and Practice of College English Speaking under the Background of Great Ideological and Political Education'', grant number 2023B2ZZ53. |
| **Funded studies**  Enter a statement with the following details:   - Initials of the authors who received each award - Grant numbers awarded to each author - The full name of each funder - URL of each funder website - Did the sponsors or funders play any role in the study design, data collection and analysis, decision to publish, or preparation of the manuscript? |  |
| Did you receive funding for this work?" |  |
| Please select the country of your main research funder (please select carefully as in some cases this is used in fee calculation). | CHINA - CN |

| as follow-up to "**Financial Disclosure**  Enter a financial disclosure statement that describes the sources of funding for the work included in this submission. Review the [submission guidelines](http://journals.plos.org/plosone/s/submission-guidelines#loc-financial-disclosure-statement) for detailed requirements. View published research articles from [*PLOS ONE*](https://journals.plos.org/plosone/) for specific examples.  This statement is required for submission and **will appear in the published article** if the submission is accepted. Please make sure it is accurate. |  |
| --- | --- |
| **Funded studies**  Enter a statement with the following details:   - Initials of the authors who received each award - Grant numbers awarded to each author - The full name of each funder - URL of each funder website - Did the sponsors or funders play any role in the study design, data collection and analysis, decision to publish, or preparation of the manuscript? |  |
| Did you receive funding for this work?" |  |
| **Competing Interests**  Use the instructions below to enter a competing interest statement for this submission. On behalf of all authors, disclose any [competing interests](http://journals.plos.org/plosone/s/competing-interests) that could be perceived to bias this work—acknowledging all financial support and any other relevant financial or non- financial competing interests.  This statement is required for submission and **will appear in the published article** if the submission is accepted. Please make sure it is accurate and that any funding sources listed in your Funding Information later in the submission form are also declared in your Financial Disclosure statement. | The authors have declared that no competing interests exist. |

| View published research articles from  [*PLOS ONE*](http://journals.plos.org/plosone/) for specific examples. |  |
| --- | --- |
| **NO authors have competing interests**  Enter: *The authors have declared that no competing interests exist.*  **Authors with competing interests**  Enter competing interest details beginning with this statement:  *I have read the journal's policy and the authors of this manuscript have the following competing interests: [insert competing interests here]* |  |
| * typeset |  |
| **Ethics Statement**  Enter an ethics statement for this submission. This statement is required if the study involved:   - Human participants - Human specimens or tissue - Vertebrate animals or cephalopods - Vertebrate embryos or tissues - Field research   Write "N/A" if the submission does not require an ethics statement.  General guidance is provided below. Consult the [submission guidelines](http://journals.plos.org/plosone/s/submission-guidelines#loc-guidelines-for-specific-study-types) for detailed instructions. **Make sure that all information entered here is included in the Methods section of the manuscript.** | The study was approved by the Academic Ethics Committee of Jiangxi Agricultural University, and the data were analyzed anonymously with consent. |

| **Format for specific study types**  **Human Subject Research (involving human participants and/or tissue)**   - Give the name of the institutional review board or ethics committee that approved the study - Include the approval number and/or a statement indicating approval of this research - Indicate the form of consent obtained (written/oral) or the reason that consent was not obtained (e.g. the data were analyzed anonymously)   **Animal Research (involving vertebrate animals, embryos or tissues)**   - Provide the name of the Institutional Animal Care and Use Committee (IACUC) or other relevant ethics board that reviewed the study protocol, and indicate whether they approved this research or granted a formal waiver of ethical approval - Include an approval number if one was obtained - If the study involved *non-human primates*, add *additional details* about animal welfare and steps taken to ameliorate suffering - If anesthesia, euthanasia, or any kind of animal sacrifice is part of the study, include briefly which substances and/or methods were applied   **Field Research**  Include the following details if this study involves the collection of plant, animal, or other materials from a natural setting:   - Field permit number - Name of the institution or relevant body that granted permission |  |
| --- | --- |
|  |  |
| **Data Availability**  Authors are required to make all data underlying the findings described fully available, without restriction, and from the time of publication. PLOS allows rare exceptions to address legal and ethical concerns. See the [PLOS Data Policy](http://journals.plos.org/plosone/s/data-availability) and [FAQ](http://journals.plos.org/plosone/s/data-availability#loc-faqs-for-data-policy) for detailed information. | Yes - all data are fully available without restriction |

| A Data Availability Statement describing where the data can be found is required at submission. Your answers to this question constitute the Data Availability Statement and **will be published in the article**, if accepted. |  |
| --- | --- |
| **Important:** Stating ‘data available on request from the author’ is not sufficient. If your data are only available upon request, select ‘No’ for the first question and explain your exceptional situation in the text box. |  |
| Do the authors confirm that all data underlying the findings described in their manuscript are fully available without restriction? |  |
| **Describe where the data may be found in full sentences. If you are copying our sample text, replace any instances of XXX with the appropriate details.**   - If the data are **held or will be held in a public repository**, include URLs, accession numbers or DOIs. If this information will only be available after acceptance, indicate this by ticking the box below. For example: *All XXX files are available from the XXX database (accession number(s) XXX, XXX.*). - If the data are all contained **within the manuscript and/or Supporting Information files**, enter the following: *All relevant data are within the manuscript and its Supporting Information files.* - If neither of these applies but you are able to provide **details of access elsewhere**, with or without limitations, please do so. For example:   *Data cannot be shared publicly because of [XXX]. Data are available from the XXX Institutional Data Access / Ethics Committee (contact via XXX) for researchers who meet the criteria for access to confidential data.*  *The data underlying the results presented in the study are available from (include the name of the third party* | All relevant data are within the manuscript and its Supporting Information files. |

| *and contact information or URL).*   - This text is appropriate if the data are owned by a third party and authors do not have permission to share the data.   * typeset |  |
| --- | --- |
| Additional data availability information: | Tick here if the URLs/accession numbers/DOIs will be available only after acceptance of the manuscript for publication so that we can ensure their inclusion before publication. |

<https://www2.cloud.editorialmanager.com/pone/download.aspx?id=39932985&guid=a38c16da-c410-4d4e-b23f-3c05374e34c8&scheme=1>title page	[Click here to access/download;Manuscript;title](https://www2.cloud.editorialmanager.com/pone/download.aspx?id=39932985&guid=a38c16da-c410-4d4e-b23f-3c05374e34c8&scheme=1)

[page20250227.docx](https://www2.cloud.editorialmanager.com/pone/download.aspx?id=39932985&guid=a38c16da-c410-4d4e-b23f-3c05374e34c8&scheme=1)

# How Does Value Integration Impact EFL Oral English Learning? Textbook Analysis and Learner Perspective

**Author:** Xixi Yang

**Institute:** School of Foreign Languages, Jiangxi Agricultural University, Nanchang, China

**Correspondence:** Xixi Yang, [1053617275@qq.com,](mailto:1053617275@qq.com) School of Foreign Languages, Jiangxi Agricultural University, Nanchang, 330045, China

**Fund:** This paper is supported by the Educational Reform Research Project of Jiangxi Agricultural University, with the grant number 2023B2ZZ53.

<https://www2.cloud.editorialmanager.com/pone/download.aspx?id=39932998&guid=454ed55e-41f3-41c8-8845-1bc8df537dda&scheme=1>Manuscript anonymous	[Click here to](https://www2.cloud.editorialmanager.com/pone/download.aspx?id=39932998&guid=454ed55e-41f3-41c8-8845-1bc8df537dda&scheme=1)

[access/download;Manuscript;20250227anonymous.doc](https://www2.cloud.editorialmanager.com/pone/download.aspx?id=39932998&guid=454ed55e-41f3-41c8-8845-1bc8df537dda&scheme=1)

# How Does Value Integration Impact EFL Oral English Learning? Textbook Analysis and Learner Perspective

**Abstract:** The research is motivated by the importance of understanding how value education is intertwined with language learning, and how this synthesis contributes to the holistic development of learners. The research employed a mixed-methods approach, which involved categorizing and analyzing verbal texts from the selected EFL oral English textbook series at the college level. According to frequency statistics, the sample textbooks include 273 verbal text samples embedded with morals and values, and a Chi-Square Test shows a significant difference of dimension emphasis between different textbook volumes. Within the theoretical framework of Martin and White's (2005) Language Appraisal Theory, a qualitative textual analysis reveals the value significance of lexical-grammatical items as a prominent feature across various teaching modules. Additionally, a questionnaire survey was conducted with EFL learners who used the textbook series for an academic year. The survey showed that values-based language instruction positively impacted morality and personal growth.

**Keywords:** integration of morals and values; textbook analysis; oral English learning; value-driven language instruction; learner outcome

# Introduction

In the educational landscape, the indispensable role of value education in shaping well-rounded individuals is increasingly recognized. This recognition is reflected in the intentional integration of morals and values into the curriculum. The infusion of values is strategic, aiming to cultivate a holistic approach to character development. Instilling ethical principles and values constitutes a fundamental component of any well-rounded discipline education.

The integration of morals and values into the curriculum is deemed essential, a viewpoint that Mangubhai (2007) supports by highlighting the moral and ethical dimensions of language teaching. This inclusion in a teacher's cognition, is seen as fundamental in influencing the learner behavior and objectives of language instruction within the classroom. The strategic moral and value integration into the curriculum is further underscored in the English Language Education Key Learning Area Curriculum Guide. This guide not only encourages the inclusion of personal and social values such as honesty, self-esteem, and perseverance but also advocates for fostering an appreciation for equality and tolerance of differences (Feng, 2017). This approach to curriculum design reflects a deliberate effort to use education as a platform for in-depth and extensive deliberation on a wide range of values, where educational materials facilitate meaningful discussions.

Textbooks are a vehicle that provides opportunities for learners to recognize and understand cultural values, including moral values, which are obeyed and used as guidelines in social life (Pratiwi, 2023). Textbooks are an important learning and teaching resource, as they have the potential to play a significant role in influencing learners' worldviews as they develop their understanding of different cultures (Lu, 2022). They go beyond transmitting subject-specific knowledge and are instrumental in nurturing moral sensitivity and behavior by including stories of role models and their behaviors, thereby significantly influencing students' moral identities (Tse & Zhang, 2017). In China, where moral education is not merely a supplementary aspect but a pivotal element intended to shape students' values, worldviews. Moral education textbooks for primary schools in China from 1999 to 2005 show an increasing emphasis on moral values such as protecting the environment, loving peace, and promoting democratic cooperation (Tse & Zhang, 2017). Sense of social responsibility, patriotism are moralized and promoted as core values in Chinese education, essential for being a good citizen (Lin & Jackson, 2023).

In 2021, the National Textbook Committee issued the *Guidelines for Incorporating Content Related to ‘Party Leadership' into Curricula for Primary, Secondary and Tertiary Schools,* marking the first time that content related to party

leadership has been integrated into the overall design of curriculum for all levels. It requires adhering to the correct values, strengthening overall design, and enhancing educational goals, fully leveraging the core function of textbooks in education. In line with national educational policies that have systematically integrated moral and value education across various curricula, the integration of values into the curriculum and textbooks is a strategic move to align educational goals with the moral and ethical standards set by the state. This approach is particularly evident in China. The *Guide for College English Teaching* (2020 edition) points out that college English teaching should proactively integrate socialist core values, explore rich humanistic connotations, guide students to establish correct views on life, values, and the world, and lay a solid moral foundation for cultivating foreign language talents. The national emphasis on moral education is a reflection of the government's commitment to cultivating responsible and morally conscious citizens who can contribute positively to society.

# Literature review

### Moral education

Moral education is fundamentally about nurturing character and values within individuals, extending beyond the classroom to encompass a broader spectrum of societal expectations and cultural norms (Bates, 2019; Wang, 2016). It has evolved to include resilience, respect for core values, and personal responsibility, now recognized as the 'new three Rs' in English education, reflecting a societal push towards producing individuals capable of enhancing national competitiveness and social mobility (Bates, 2019). This aligns with the broader goal of moral education globally, which aims to educate people to become good citizens in both knowledge and morality (Phan & Phan, 2006; Bates, 2019). Moreover, the continuous process of moral development is emphasized, suggesting that value education is not confined to formal institutions but is a lifelong journey that begins at home and extends into society (Prahaladaiah, 2021). Saldivar et al. (2024) found that personal values such as achievement, self-direction, security, and hedonism significantly influence international students’ university selection criteria. This highlights the importance of moral education in shaping individuals' decisions, even in the context of higher education.

The historical narrative of moral education is deeply rooted in the evolution of state ideologies and the propagation of societal values. In Korea, the establishment of moral education as an independent subject in 1973 was a response to the nationalistic sentiments stirred by the Korean War and the ongoing tensions between the North and

South, leading to the emergence of values such as patriotism and the will for unification that continue to influence the moral education curriculum (Han et al., 2018, as cited in Roh, 2004b). This mirrors the cultural approach to moral education seen in Japan with the introduction of Dotoku in 1958, emphasizing the path of virtue, and in China since 1949, where moral education has been inextricably linked with political ideologies, implementing extensive ideological education programs across all social sectors, including the schooling system (Han et al., 2018, as cited in Khan, 1998; Tse & Zhang, 2017). These historical contexts highlight how moral education has served as a tool for instilling national identity and promoting specific societal values. China's moral education curriculum contains competing ideas about the state-citizen relationship, with some values emphasizing the protection of individual freedoms and rights, while others prioritize the citizen's duty to contribute to and sacrifice for the country (Liu, 2023).

Value education is crucial for fostering good citizenship and social cohesion. Textbooks and educational programs, such as ESOL in the United States, play a significant role in educating individuals to become good citizens, focusing on didactic education and exposing learners to cultural values and behaviors (Feng, 2017; Pentón Herrera, 2019). This approach is further supported by the argument that cultural content in English language teaching materials should reflect the diversity of English as an international language, catering to learners' communication needs and their own cultural contexts (Rahim & Daghigh, 2020). Additionally, value education is highlighted as a critical component in negotiating multifaceted identities in English classes, where native oriental culture and philosophical values are deeply embedded (Gao, 2020). This underscores the role of moral education in cultural identity formation and the broader implications for global citizenship. Teachers play a pivotal role in this process, serving as moral guides or role models, and their investment in student development encompasses both the sociopolitical and the educational, shaping their behaviors in the classroom (Briggs & Kim, 2020; Kusramadhani et al., 2022). Coleman and Leider (2013) highlight the importance of teachers positioning themselves as both educators and learners in the classroom. Their self-study demonstrates that teachers can empower themselves and their students by engaging in reflective practices and curriculum design that promote personal and professional growth. These concerns highlight the importance of value input in teaching practices (Mangubhai, 2007).

### Other important values imparted through educational approaches

Global educational initiatives are increasingly recognizing the significance of personal development and well-being, with a focus on cultivating healthy lifestyles,

positive energy, and self-confidence. Textbooks and curricula promote values such as good hygiene, regular exercise, balanced diets, and self-regulation to encourage students to take responsibility for their well-being (Feng, 2017; Pratiwi et al., 2023). Meanwhile, programs that emphasize autonomy, resilience, and social-emotional skills aim to enhance students' self-motivation and overall well-being by encouraging active participation in community service and personal development (Bates, 2019; Han et al., 2018; Mangubhai, 2007). Collectively, these initiatives contribute to building confident and self-aware individuals who are capable of taking initiative in their learning and social interactions.

A variety of educational materials and programs are playing a crucial role in fostering environmental and social responsibility among students. Textbooks and curricula highlight the importance of nature conservation, respect for life, and sustainable practices to cultivate a sense of responsibility and care for the environment (Han et al., 2018; Pratiwi et al., 2023). In addition, values such as respect, tolerance, and community engagement are emphasized to enhance students' understanding of social issues and their role in contributing to the greater good (Bates, 2019; Keskin, 2013; Mangubhai, 2007). These initiatives aim to develop students' appreciation for the natural world and foster a sense of compassion and responsibility towards all living beings.

Educational programs worldwide are focusing on developing professional and cultural competence by emphasizing values such as responsibility, respect, law-abidingness, and cultural heritage. Curricula aim to prepare students for future careers by fostering essential social and emotional skills, promoting a sense of purpose, and encouraging personal development (Bates, 2019; Han et al., 2018; Keskin, 2013; Pratiwi et al., 2023). Furthermore, educational initiatives that emphasize independence and the pursuit of excellence aim to empower students to become autonomous learners and contributors to society (Liu, 2023; Ministry of Education, 2017). Collectively, these programs contribute to developing students' cultural awareness and preparing them to be global citizens.

Educational initiatives are fostering national and cultural pride by emphasizing values such as respect, responsibility, and national identity. These initiatives aim to enhance students' self-esteem and sense of belonging by encouraging them to take pride in their cultural heritage and contributions to society (Han et al., 2018; Liu, 2023; Pratiwi et al., 2023).

### Textbook representation

Textbooks play a central role in EFL education, serving as the primary carrier of social values and taking on multiple roles in cultural teaching, including being a teacher, map, resource, trainer, and an authority and ideology (Feng, 2017; Song, 2019). Their influence extends to shaping teacher beliefs and professional development within educational contexts. The transition towards global textbooks in Malaysia, which has seen the replacement of locally developed textbooks with imported ones, raises questions about their ability to convey local values and underscores challenges in promoting a national identity curriculum due to the increasing tension between local autonomy and national cohesion (Rahim & Daghigh, 2020; Ye, 2022). Additionally, textbooks are instrumental in implementing character education policies, as demonstrated by their use in Indonesia to inculcate values and shape student behavior (Qoyyimah, 2016).

The intentional construction of cultural and moral values within EFL textbooks is a complex process that requires a comprehensive approach, including various linguistic, rhetorical, and multimodal resources (Xiong, 2012; Li, 2023). This is underscored by initiatives like the Australian Values Education Program, which highlights moral development as a core educational goal, and the cultural specificity of values, such as the unique emphasis on morality in Chinese culture (Lovat, 2017; Meyer, 1988). The integration of values into the curriculum, as seen in the Indonesian EFL textbook "When English Rings a Bell," and the negotiation of teacher identity with regard to morality in non-Western contexts, such as how non-Western teachers negotiate their identity with regard to morality, complicates the representation of values in EFL materials (Kusramadhani et al., 2022; Phan & Phan, 2006).

Research provides empirical evidence of the characteristics of moral exemplars in EFL textbooks, emphasizing the importance of diverse representations and a balanced approach to cultural teaching (Han et al., 2018; Song, 2019). The value dimension of English language teaching, explored through educators' reflections and the challenges faced by ELT teachers in engaging with value teaching practices, highlights practical challenges and ethical considerations (Briggs & Kim, 2020; Soleimani & Lovat, 2019). The ideological nature of language textbooks, representing the dominant culture and values, particularly in the context of Chinese as a foreign language education, is a key aspect of this discourse (Wang, 2016). Furthermore, the importance of exploring moral values and behavior through the experiences of English learners in ESOL classrooms is emphasized, suggesting a student-centered approach to moral education (Pentón Herrera, 2019).

While prior research has examined the integration of morals and values into EFL materials and the complexities of cultural representation, empirical studies specifically targeting oral English textbooks and the particular morals and values they convey remain sparse. Moreover, the learner's perspective on the moral values

acquired through oral English instruction in an EFL setting has yet to be thoroughly explored. To address these identified gaps, the current study seeks to investigate the following research questions:

Question 1: What are the primary morals and values incorporated in EFL oral English textbooks, and how are they integrated and presented?

Question 2: How do different volumes within the same EFL oral English textbook series vary in their emphasis on morals and values, and how do these differences reflect distinct educational objectives or learner needs?

Question 3: What morals and values do learners gain from EFL oral English classes, and how do these acquisitions impact their language learning process and personal growth?

# Appliable linguistics framework for analyzing verbal texts

The Language Appraisal Theory, as developed by Martin and White (2005), offers a comprehensive approach to understanding how attitudes are expressed through language. This framework categorizes attitudes into three main domains: affect, judgement, and appreciation, each with its own subcategories that allow for a nuanced exploration of emotional responses, assessments of character and behavior, and evaluations of objects and performances.

Affect captures the emotional response of the speaker or writer and is divided into un/happiness, in/security, and dis/satisfaction. These subcategories reflect a range of emotions from joy and sadness to feelings of safety, confidence, and the fulfillment of desires or needs. Judgement involves the assessment of actions, behaviors, or character traits. It is further divided into social esteem, which includes normality (how usual), capacity (how capable), and tenacity (how resolute), and social sanction, encompassing veracity (how truthful) and propriety (how ethical). These categories evaluate the social status, acceptability, morality, ethics, and reliability of individuals or groups. Appreciation pertains to the evaluation of objects, phenomena, or performances and is divided into reaction (impact and quality), composition (balance and complexity), and valuation (worth). This category assesses the impact, structure, organization, and worth or value of entities within specific contexts. The framework allows for a detailed analysis of how speakers or writers convey their feelings, assessments, and evaluations in various contexts.

# Mixed methods

### Categorizing morals and values in textbooks

*New Inside Out* is an ideal EFL teaching resource for moral education research due to its diverse and relevant content, comprehensive language skills learning, and cultural richness. Its widely accepted use in higher education makes it a valuable tool for investigating value education outcomes. The ongoing updates to its content provide a dynamic perspective for research. To address the first research question, the researcher began by categorizing the verbal texts in *New Inside Out* Book 1 and Book 2, which are used as the first-year oral English textbooks in a university in mid China. In the text analysis, the researcher focused on verbal texts that convey values through dialogues, listening, passage reading, writing, grammar work, vocabulary exercise and other related texts. By reading through a total of 318 pages of the two textbook volumes, I tried to find the morals and values embedded in the texts. A general quantitative analysis was conducted to determine the specific value types displayed from the original verbal texts and recorded the data in an Excel sheet. Using attitude coding, I calculated the occurrence and frequency of the 27 types of values represented in the textbooks. This multi-dimensional analysis aimed to uncover the nuanced ways in which the textbooks promote and reinforce values, thereby highlighting the interconnections between language learning, value shaping, and personal growth.

### Chi-Square Test for analyzing value distribution in textbooks

To answer the second research question, the frequency data of value elements from the textbook series was extracted and input into SPSS 26 for a Chi-Square Test analysis. The objective was to identify any significant differences in the distribution of values across different volumes of the textbook series, thereby revealing the specific moral and value elements emphasized in Book 1 and Book 2. However, due to the dilution of data caused by the dispersed frequencies across the 27 moral types, the first attempt was unsuccessful. The low expected counts in 68% of the cells led to an underpowered test, which failed to detect significant differences between the textbooks. Therefore, the 27 value types were reorganized into five broader dimensions based on their conceptual correlations (as categorized in Table 1). The researcher then aggregated the frequencies within each dimension and conducted a second Chi-Square Test. The second attempt produced a more favorable result, as the test statistics (Pearson Chi-Square, Likelihood Ratio and Fisher's Exact Test) indicated significant differences in the distribution of moral dimensions between the

textbooks being compared (p < .05). This suggests that the textbooks have distinct emphases in their value contents.

## Table 1

Value dimensions.

## Emotional and Social Relationship

Caring and love Respect Kindness and friendliness Empathy Tolerance

## Ethical and Moral Principles

Responsibility Rule awareness Honesty and trust

Thrift Fairness Hard work Discipline Integrity

## Personal

**Well-being and Career Development**

Love for the healthy lifestyle

Positive energy Pride

Career ideal Independence Confidence Professionalism Pursuit of excellence

## Innovation and Social-Cultural Sensitivity

Social awareness Creativity Cultural awareness

## Eco-consciousness and Compassion

Environmental awareness Admiration for nature

Care for animals

### Online survey collection for learner outcomes

For the third research question, the researcher used the online survey platform Wen Juan Xing (WJX) to create a questionnaire to gather learner feedback on effectiveness of the value education in the oral English course. Before the survey began, all participants were informed about the purpose, procedures, potential risks, and the intended use of the survey data. They were also assured that their participation was voluntary, and they had the right to withdraw at any time without any negative consequences. All participants provided informed consent prior to their involvement in the survey. Since this was an online-based survey, written informed consent was obtained in the form of an electronic agreement. When participants accessed the questionnaire on the WJX platform, they were presented with an informed consent statement at the beginning. By clicking "I agree" and proceeding to the questionnaire, they indicated their written consent.

The survey was conducted from April 18 to May 31, 2023, with weekly reminders sent to participants to improve response rates. The questionnaires were distributed to a sample of the freshmen, who have used the textbook series for one academic year. To eliminate bias, and ensure truthful responses, the questionnaire was conducted anonymously. This safeguarded participant privacy and ensured reliable and valid data. Survey data were obtained from WJX database on June 9, 2023. All

responses were de-identified to protect participant confidentiality, and no personally identifiable information was retained. A total of 140 questionnaires were distributed initially. A total of 154 responses were collected via WJX, with the surplus resulting from multiple submissions originating from shared IP address. Three students did not submit the questionnaire according to the learning platform statistics. After comparing the response time, 16 questionnaires were deemed invalid due to a completion time of less than 35 seconds. Such short completion times suggest that respondents may not have engaged sufficiently with the questions, which could lead to inaccurate or unreliable data. Excluding these ensures that the data set consists of thoughtful and attentive responses. Ultimately 135 valid questionnaires were collected, resulting in a response rate of 96.43%.

# The results and analysis

### Key value themes in oral English textbook series

**Fig. 1.** Value elements distribution in the textbook series.

Based on a thorough analysis of the value integration in the *New Inside Out* Book 1 and Book 2, the researcher found 27 types of values contained in 273 verbal text samples out of 318 pages, ranging from "professionalism" to "caring and love"(see Fig. 1.). Certain value elements occupy significant positions in the textbooks. Specifically, "caring and love" stands out as the most prominent value, with 35 occurrences and a percentage of 12.82%. "Love for the healthy lifestyle" follows closely with 32 occurrences and a percentage of 11.72%. "Respect", "rule awareness", and "kindness and friendliness" rank third to fifth, with 20, 17, and 15 occurrences, and percentages of 7.33%, 6.23%, and 5.49%, respectively. Next in line, "environmental awareness" holds the sixth position with 19 occurrences, reflecting a 6.96% emphasis within the textbooks. Following closely, "responsibility" ranks seventh, noted 17 times and accounting for 6.23% of the total. "Positive energy" ranks eighth, with 14 occurrences, which accounts for 5.13%, which is the final one that exceeds 5% in percentage.

### Textbook volume disparity in value representation

| **Table 2**  Case Processing Summary | . |  | | | | |
| --- | --- | --- | --- | --- | --- | --- |
|  |  | Valid |  | Missing |  | Total |
|  | N | Percent | N | Percent | N | Percent |
| Dimensions | 273 | 100.0% | 0 | 0.0% | 273 | 100.0% |

* Book

## Table 3

Value Dimensions * Book Crosstabulation.

## Value Dimensions * Book Crosstabulation

Book

|  | | | book1 | book2 | Total |
| --- | --- | --- | --- | --- | --- |
| Value | Ethical and Moral | Count | 22a | 38a | 60 |
| Dimensions | Principles | Expected Count | 27.3 | 32.7 | 60.0 |
|  |  | % within Book | 17.7% | 25.5% | 22.0% |
|  | Innovation and | Count | 15a | 16a | 31 |
|  | Social-Cultural | Expected Count | 14.1 | 16.9 | 31.0 |
|  | Sensitivity | % within Book | 12.1% | 10.7% | 11.4% |
|  | Emotional and Social | Count | 23a | 59b | 82 |
|  | Relationship | Expected Count | 37.2 | 44.8 | 82.0 |

|  |  | % within Book | 18.5% | 39.6% | 30.0% |
| --- | --- | --- | --- | --- | --- |
|  | Eco-consciousness | Count | 17a | 11a | 28 |
|  | and Compassion | Expected Count | 12.7 | 15.3 | 28.0 |
|  |  | % within Book | 13.7% | 7.4% | 10.3% |
|  | Personal Well-being | Count | 47a | 25b | 72 |
|  | and Career | Expected Count | 32.7 | 39.3 | 72.0 |
|  | Development | % within Book | 37.9% | 16.8% | 26.4% |
| Total |  | Count | 124 | 149 | 273 |
|  |  | Expected Count | 124.0 | 149.0 | 273.0 |
|  |  | % within Book | 100.0% | 100.0% | 100.0% |

Each subscript letter denotes a subset of Book categories whose column proportions do not differ significantly from each other at the .05 level.

## Table 4

Chi-Square Tests.

Monte Carlo Sig. (2-sided)

Asymptotic

99% Confidence Interval

|  | Value | Df | Significance  (2-sided) | Significanc  e | Lower  Bound | Upper  Bound |
| --- | --- | --- | --- | --- | --- | --- |
| Pearson | 26.041^a^ | 4 | .000 | .000^b^ | .000 | .000 |
| Chi-Square |  |  |  |  |  |  |
| Likelihood Ratio | 26.541 | 4 | .000 | .000^b^ | .000 | .000 |
| Fisher's Exact | 26.208 |  |  | .000^b^ | .000 | .000 |
| Test |  |  |  |  |  |  |

N of Valid Cases	273

a. 0 cells (0.0%) have expected count less than 5. The minimum expected count is 12.72.
b. Based on 10000 sampled tables with starting seed 2000000.

**Fig. 2.** Value dimension frequency.

The distribution of value dimensions between Book 1 and Book 2 was examined using a Chi-Square Test (Table 2, Table 4). The Pearson Chi-Square Test yielded a significant value of 26.041 with 4 degrees of freedom (p = .000), indicating a notable difference in the moral education focus between the two textbook volumes at the 0.05 significance level. This was further corroborated by the Likelihood Ratio Test, which also showed a significant value of 26.541 (p = .000). To address potential concerns about small expected counts, Fisher's Exact Test was conducted, confirming the significant difference with a value of 26.208 (p = .000). All cells in the contingency table had expected counts exceeding 5, with the minimum being 12.72, thereby satisfying the assumptions necessary for the chi-square test.

Upon detailed examination of the crosstabulation results (Table 3), it is evident that the emotional and social relationship dimension and the personal well-being and career development dimension are the primary areas of distinction between Book 1 and Book 2 (see Fig.2.). The subscript letters 'a' and 'b' in the table differentiate between subsets of Book categories within a dimension, where categories sharing the same letter are not significantly different from each other. The personal well-being and career development dimension is markedly more prevalent in Book 1, which includes 47 instances (37.9%), in contrast to Book 2's 25 occurrences (16.8%).

Similarly, the emotional and social relationship dimension is particularly emphasized in Book 2, with 59 occurrences (39.6%) compared to Book 1's 23 occurrences (18.5%). The remaining dimensions—ethical and moral principles, innovation and social-cultural sensitivity, and eco-consciousness and compassion—do not display significant differences between the textbook series when considering the post hoc comparisons. The shared subscript letter 'a' across these dimensions indicates that they belong to the same subsets at the .05 significance level, suggesting similar emphasis or lack of significant variation in how these dimensions are addressed in both textbooks.

### Value threads in EFL textbook narratives

In this section, the researcher looks at the narrative analysis of the representation of the top eight values, with a percentage of above 5% each. These dominant values of the two textbook volumes are as follows: "care and love", "love for the healthy lifestyle", "respect", "environmental awareness", "responsibility", "rule awareness", "kindness and friendliness", and "positive energy". These values are prominently featured across various modules in the textbooks. The samples provided below are drawn from different units and sections, including Listening, Speaking, Reading, Writing, Grammar and Vocabulary.

1) Caring and love

Text 1: Listening. I didn't want to lose him again! (Book 1 Unit 3 p20)

Text 2: Speaking. What's the best present you've ever given or received? (Book 1 Unit 4 p32)

Text 3: Reading. I keep this photo on my desk at work, because it makes me smile every time I look at it. It's a photo of my wife. (Book 2 Unit 3 p20)

Text 4: Grammar. You don't look very happy. Has something happened at work? (Book 2 Unit 4 p35)

Text 5: Reading & Vocabulary. A mum is a person who cares for you and tucks you in at night. (Book 2 Unit 10 p88)

The statement "I didn't want to lose him again!" in text 1(affect-dis/satisfaction) conveys a strong sense of dissatisfaction with the potential loss, reflecting an emotional attachment and the distress that comes from the possibility of losing someone cherished. This highlights the depth of the speaker's desire to maintain the relationship and the dissatisfaction that the threat of separation brings. This observes

that EFL textbooks often represent social values through explicit and implicit judgments, including emotional responses like happiness, satisfaction, and distress (Feng, 2017). "Best present" in text 2 (appreciation-valuation) is a valuation of an object or gift, signifying an assessment of its worth in terms of personal experience or emotional value. Text 3 (affect-un/happiness) reflects a positive emotional response and a source of happiness, which is a subtle indication of the love and care presented in the relationship. Text 4's (judgement-social esteem-normality) inquiry about the individual's appearance and mood could be interpreted as a judgment about the normality of their behavior or emotional state. The speaker seems to recognize that the individual's current demeanor deviates from what is typically expected, prompting an inquiry that acknowledges this deviation and shows care for their well-being. Text

4 reinforces teacher's role as a moral guardian and caregiver, highlighting the importance of understanding students' emotions and supporting their psychological development (Qoyyimah et al., 2023). It also discusses the moral dimension where teachers are expected to be aware of and respond to students' emotional and psychological states (Briggs & Kim, 2020). The actions of tucking in at night and caring in text 5 (judgement-social esteem-tenacity) could be seen as indicative of a mother's tenacity and commitment to providing a sense of safety and love, which is a form of social esteem for the consistent and enduring nature of a mother's care.

2) Love for the healthy lifestyle

Text 6: Listening & Speaking. We all know the expression ''laughter is the best medicine''. (Book 1 Unit 5 p45)

Text 7: Grammar. A mother to her teenage son: ''You shouldn't watch so much television. It's bad for you.'' (Book 1 Unit 8 p73)

Text 8: Reading & Vocabulary. She gets up at 7.00 a.m. every day and goes for a brisk walk. Three times a week she plays gate-ball (a popular national sport) with her friends. There is nothing unusual about this, except that Chiako is 102 years old. (Book 1 Unit 10 p88)

Text 9: Reading. I think a happy childhood gives you balanced view of food and yourself. I eat what I fancy and feel good about it. I feel lucky that I escaped all the size zero nonsense when I was younger. (Book 2 Unit 5 p44)

The use of the word "best" in text 6 (Appreciation-valuation) indicates a positive assessment of the value of laughter in comparison to other forms of medicine or therapy. In text 7 (judgement-social sanction-propriety) the mother's advice to her son about watching less television implies a judgment on the propriety of spending excessive time on this activity, suggesting that it is not conducive to a healthy lifestyle.

This advice supports the broader educational goal of promoting healthy lifestyles through self-regulation and balanced habits, as highlighted in curricula and programs aimed at personal development (Feng, 2017; Pratiwi et al., 2023). Text 8 (judgement-social esteem-tenacity) shows Chiako's daily routine of waking up early and engaging in physical activities, despite her age. Her commitment to these activities at the age of 102 is exceptional and shows a strong sense of determination and perseverance, which are key aspects of tenacity. In text 9 (affect-un/happiness) the speaker expresses a positive emotional state (feeling good) and a sense of luck, which are both indicators of happiness.

3) Respect

Text 10: Reading. What's the best way to make a really good impression at a party? By wearing the best clothes? Telling the best jokes? Dancing like a professional? No --you just need to remember people's names. (Book 1 Unit 1 p7)

Text 11: Reading & Listening. Your friend is going to meet his girlfriend's parents for the first time. Look at the advice(a-f). c)Wear the right clothes (Book 1 Unit 8 p72)

Text 12: Speaking & Reading. Don't worry about the mess! It's no fun for party guests to see the host running around holding a dustpan and brush. (Book 2 Unit 4 p36)

Text 13: Listening b) It's OK/not OK when paparazzi take unflattering photos of celebrities. (Book 2 Unit 7 p60)

Text 14: Speaking. My mother plays the piano really well, so she was very keen for me to learn the piano too. I had lessons for a while, but I was useless, and eventually my piano teacher begged my parents to stop sending me. So that was the end of my music career! But what I really wanted to do was martial arts, and my parents let me join a club when I was about nine. (Book 2 Unit 10 p93)

Text 10 (Judgement-social esteem-normality) is discussing what is considered a norm or a common practice in social interactions—that remembering people's names is a valued and effective way to make a good impression. Text 10 does resonate with the broader theme of recognizing and valuing individuals, which is demonstrated in the argument that "everyone has worth" (Mangubhai, 2007). This act of recognition can lead to positive emotions and strengthen social bonds, which is a fundamental aspect of moral education and interpersonal relationships. Wearing the right clothes in text 11 (judgement-social sanction-propriety) is advised when meeting someone's parents for the first time, which indicates a judgment about the appropriateness of

attire, reflecting social acceptability and respect for the occasion. In text 12 (judgement-social sanction-propriety) the advice not to worry about the mess and not to clean up in front of guests suggests a judgment on the propriety of hosting behavior. It implies that creating a relaxed atmosphere is more respectful to guests than obsessing over cleanliness. This emphasizes the importance of respecting others and their cultural norms, highlighting the significance of this value in fostering positive social interactions (Keskin, 2013). The statement in text 13 (judgement-social sanction-propriety) about paparazzi taking unflattering photos of celebrities touches on the propriety of such actions, questioning the ethics and respect for privacy and personal boundaries. In text 14 (affect-dis/satisfaction), to everyone's dissatisfaction, the mother's keen interest in the speaker learning piano didn’t work out. The speaker uses the adjective "useless" to describe his failure. However, the speaker's real passion lies in martial arts, which the parents eventually support. This echoes the perspective that adults' values, whether parents or teachers, influence the learning climate, impacting students' personal growth and behavior (Soleimani & Lovat, 2019).

4) Environmental awareness

Text 15: Reading. A spokesman from the Green Party said, ''We are not against fashion, but cheap clothes, not designed to last, are bad for the consumer and the planet.'' (Book 1 Unit 4 p37)

Text 16: Listening. Debbie, 37. I'm protesting against multinational companies. They're polluting our rivers and oceans and they're causing global warming. I'm in favour of small family-run companies. I'm against food imports. I support local farmers and I buy food from farmers' markets, not supermarkets. (Book 1 Unit 7 p61)

Text 17: Reading & Vocabulary. Then imagine white sands and coral gardens never damaged by dynamite fishing or trawling nets. (Book 2 Unit 8 p69)

Text 18: Useful phrases.

Kim: Don't you think bicycles are dangerous?

Ryan: Not really. They are certainly less dangerous for the environment. (Book 2 Unit 9 p82)

The Green Party spokesman's statement in text 15 (judgement-social esteem-capacity) critiques "cheap clothes" for not being designed to last, suggesting a lack of consideration for environmental impact by the producers. This reflects a judgment on their capacity to contribute positively to sustainability. The critique of non-durable clothing is in line with the character education policy's aim to instill "environment awareness" among students, a value which is noted as part of the curriculum

(Qoyyimah, 2016). This critique supports the broader educational goal of fostering environmental responsibility and sustainable practices among students, as emphasized in various educational materials and programs (Han et al., 2018; Pratiwi et al., 2023). Text 16 (judgement-social sanction-propriety) demonstrates her disapproval of food imports and supermarket purchases, arguing that they are ethically and socially inappropriate due to their environmental impact. Text 17 (appreciation-reaction) evokes an emotional reaction by imagining pristine white sands and coral gardens unaffected by destructive fishing practices, appreciating the untouched natural beauty and its value. This appreciation supports the broader educational goal of developing students' admiration for nature and fostering a sense of compassion and responsibility towards the environment, as highlighted in various educational initiatives (Han et al., 2018; Pratiwi et al., 2023). Ryan's response in text 18 (judgement-social esteem-capacity) underscores the environmental benefits of bicycles as a more sustainable and eco-friendly transportation option. This viewpoint aligns with the overarching educational objectives of cultivating environmental stewardship and promoting sustainable practices, which are integral to many contemporary educational curricula and materials (Han et al., 2018; Pratiwi et al., 2023)..

5) Responsibility

Text 19: Listening. I've been running a bar on the beach. It was difficult at first, because I opened my bar just two months before the tsunami in December 2004. Our bar was OK, but tourists stayed away for a long time after that, and I almost had to close the bar. But I've had a lot of support from my husband, and now the bar is going very well (Book 1 Unit 9 p80)

Text 20: Speaking. My mother did most of the cooking, but we all helped her. Well, sort of helped her. We each had a special job: I used to chop vegetables, my brother helped with the washing up, and my sister set the table. (Book 2 Unit 5 p45)

Text 21: Grammar. People have accused the fashion industry of encouraging young girls to go on starvation diets. (Book 2 Unit 7 p63)

The speaker in text 19 (judgement-social esteem-tenacity) acknowledges the significant support from her husband, which was crucial during the challenging times following the tsunami. This highlights the husband's resilience and commitment to the family's well-being, demonstrating a strong sense of familial responsibility and partnership. It echoes the broader context that moral education aims to develop individuals who are responsible for their actions and can contribute positively to society (Asif et al., 2020), through moral messages conveyed by the writer (Kusramadhani et al., 2022). This trait is also agreed by Text 20 (judgement-social

esteem-capacity) describes a family dynamic where each member has a "special job" in helping with cooking tasks. This allocation of roles demonstrates the family members' capacity to contribute and take responsibility for their part in the household chores. The representation of everyday roles and responsibilities within a family context is often featured by ordinary people as moral exemplars in moral education textbooks (Han et al., 2018). This approach makes moral values more relatable and attainable for students, encouraging them to emulate such behaviors in their own lives. Text 21 (judgement-social sanction-veracity) references an accusation against the fashion industry, suggesting that the speaker believes the industry is not being truthful about the potential harm to young girls' health, reflecting a lack of social responsibility.

6) Rule awareness

Text 22: Reading. I had to do my homework every night and I could only watch the television at the weekend. (Book 1 Unit 9 p70)

Text 23: Listening.

S: Fasten your seatbelt, grandad. G: But I’m sitting in the back.

S: Yes, and you have to fasten your seatbelt in the back now.

G: Oh dear, all these rules and regulations. In my day, we didn't have to wear seatbelts at all. (Book 1 Unit 9 p71)

Text 24: Useful phrases. Teacher: Come along children. Stop running. OK, one by one. Ruben, stop pushing. (Book 2 Unit 6 p54)

Text 25: Reading & Listening. Schoolgirl, Pauline Gates, has not been allowed back into school after the summer holidays because she has had her nose pierced. According to headmistress, Jean Bradley, Paula knew that piercing was against the school rules. The girl will be allowed back into school when she removes the offending nosering. (Book 2 Unit 7 p64)

In text 22 (judgement-social esteem-normality) the speaker's routine of doing homework every night and watching television only on weekends reflects a normative behavior expected in a student's life. This demonstrates an acceptance of and adherence to a schedule that balances responsibilities and leisure activities, aligning with societal expectations of discipline and time management. The dialogue between the grandfather (G) and the speaker (S) in text 23 (judgement-social sanction-propriety) highlights the grandfather's resistance to the new rule of fastening

seatbelts in the back seat. The speaker's insistence on following the rule, despite the grandfather's nostalgic reminiscence, underscores the societal value placed on safety and the ethical obligation to comply with current regulations. Text 24 (judgement-social sanction-propriety) shows that the teacher's instructions to the children to stop running and pushing reflect a judgment on the appropriateness of behavior, especially in a school setting. The use of commands by the teacher indicates a clear expectation for students to adhere to rules that ensure order and safety. Text 25 (judgement-social esteem-normality) describes a situation involving Pauline Gates, who has had her nose pierced against school rules, is an example of a deviation from the normative behavior expected of students. The headmistress's decision to not allow her back into school until the nose ring is removed reinforces the societal esteem for following established rules and maintaining a certain standard of appearance in educational institutions.

7) Kindness and friendliness

Text 26: Speaking. He's important to me because he knows me so well. When I'm feeling down or when I need to talk to somebody, I can always call him. (Book 1 Unit 1 p9)

Text 27: Reading & Speaking. There's the listener/ response smile. When two people are having a conversation, the listener smiles to encourage the speaker. (Book 1 unit 9 p76)

Text 28: Speaking & Reading. And don't forget to stock up on the chopped carrots to keep the vegetarians happy. (Book 2 Unit 4 p36)

Text 29: Reading & Speaking. Do you find it painful to share your chocolate with other people? (Book 2 Unit 5 p40)

Text 26 (judgement-social esteem-capacity) conveys that when in need, the speaker's turning to a friend hints at the underlying kindness in their relationship, suggesting a bond that provides comfort and understanding. The listener's smile in text 27 (appreciation-reaction) is a reaction to the speaker's communication, intended to have a positive impact and encourage further dialogue. Text 28 (judgement-social esteem-normality) mentions catering to vegetarians by having chopped carrots implies a social norm of consideration, subtly indicating an environment where kindness and respect for others' choices are valued. Text 29 reflects the perspective on intercultural teaching by demonstrating the importance of exposing students to diverse cultural practices, fostering an inclusive attitude, and promoting cultural empathy (Song, 2019). The question in text 29 (affect-un/happiness) implies a potential emotional

response to the act of sharing, which could range from happiness in sharing to pain or discomfort in parting with one's chocolate.

8) Positive energy

Text 30: Reading & Vocabulary. Near a beach, there is a large stone with the following proverb engraved on it: "At 70 you are still a child; at 80 you are just a youth; and at 90, if the ancestors invite you into heaven, ask them to wait until you are 100, and then you might consider it. " (Book 1 Unit 10 p88)

Text 31: Grammar. I wish I had been less anxious. (Book 2 Unit 11 p99)

Text 30 (affect-un/happiness) conveys a positive perspective on aging, suggesting a sense of joy and optimism about life at advanced ages. The proverb engraved on the stone promotes a view of aging that is not typically associated with negative emotions, but rather with a continued sense of youthfulness and potential. Similarly, the positive perspective on aging in text 30 reflects that character education promotes psychological well-being (Bates, 2019). This perspective supports the broader educational goal of fostering positive energy and self-confidence among students, as emphasized in various educational initiatives aimed at personal development and well-being (Feng, 2017; Pratiwi et al., 2023). The expression of a wish to have been less anxious in text 31 (affect-in/security) implies a reflection on past emotions that may have detracted from the speaker's overall sense of well-being. While the statement acknowledges a feeling of insecurity, it also carries a positive energy by indicating a desire for a more relaxed approach to life's challenges. It also highlights the value of self-reflection and personal growth, which contribute to a more fulfilling and meaningful life (Asif et al., 2020). This reflection aligns with educational programs that emphasize self-regulation and social-emotional skills, aiming to enhance students' overall well-being and self-motivation (Han et al., 2018; Mangubhai, 2007).

### Learner attitudes and preferences on value integration

The questionnaire consists of three parts. Part one includes basic information about the survey participants, such as their grade and gender. Part two consists of rating scale questions, which assess students' attitudes towards the integration of values in the oral English course (Table 5). Part three includes multiple-choice questions, primarily focusing on the implementation and impact of value-bssed oral English instruction.

The results of the attitude part were interpreted on the basis of the four-point Likert scale (1-4): high level (3.5-4.0), above medium level (2.5-3.4), below medium level (1.5-2.4) and low level (1.0-1.4).

| **Table 5**  Learner Perception of Value Integration. |  | |
| --- | --- | --- |
| **Questions** | **Mean** | **Level of Perception** |
| 9.Do you think value integration would help | 2.98 | Above Medium |
| stimulate interest in learning? |  |  |
| 10.Do you think value integration is beneficial for improving speaking skills? | 2.96 | Above Medium |
| 11.Do you think value integration is beneficial for students' personal growth? | 3.08 | Above Medium |
| 12. Do you think teaching morals and values in oral English positively influences students' beliefs | 3.21 | Above Medium |
| and actions? |  |  |
| There is a predominantly positive perception | among | participants regarding the |

integration of value education into learning. The average scores for the questions hover at the above medium level, with the highest score of 3.21 indicating a strong positive role of moral and value education in fostering students' values and beliefs. The scores for questions related to stimulating interest in learning (2.98) and improving speaking skills (2.96) suggest a more tempered view in these areas. However, the integration is seen as beneficial for personal growth (3.08), which is above medium level. These findings underscore the value placed on moral education as a component of a well-rounded educational experience.

Question 5: In oral English classes, which of the following topics are involved? (Multiple choices allowed)

## Table 6

Value Education Content Prioritized by Learners.

| **Options** | **Frequency** | **Percentage** |
| --- | --- | --- |
| Moral qualities | 112 | 84.21% |

| Sense of right and wrong | 104 | 78.20% |
| --- | --- | --- |
| Life and health | 98 | 73.68% |
| Patriotism | 97 | 72.93% |
| Traditional culture | 92 | 69.17% |
| Family values | 90 | 67.67% |
| Professional conduct | 77 | 57.89% |
| Environmental protection | 72 | 54.16% |
| Historical anecdotes | 68 | 51.13% |

The survey conducted on moral issues and personal development content (Table 6) in the oral English course reveals insightful trends regarding the values prioritized by participants. The top three values identified are moral qualities (84.21%), sense of right and wrong (78.20%), and life and health (73.68%) . These results indicate a strong emphasis on ethical behavior and national pride among respondents, suggesting that these themes are crucial in shaping values. In the middle range, we find patriotism (72.93%), traditional culture (69.17%) and family values (67.67%). These values highlight the importance of personal well-being and familial relationships, reflecting a balanced approach to moral education that encompasses both individual and collective aspects. On the lower end of the spectrum, values such as professional conduct (57.89%), environmental protection (54.16%), and historical anecdotes (51.13%) received comparatively less emphasis.

In Question 7 about learners' preferred teaching methods for value education by oral English teachers, the most popular choices were video watching (75.97%), topic discussion (65.58%), role-playing (61.69%), and theme-based speeches (42.21%). These engaging activities not only increased learners' interest and participation in oral English classes but also encouraged critical thinking on real issues, yielding positive moral outcomes.

Regarding changes observed after implementing value education in oral English classes, the top four responses were: rationally viewing cultural differences between China and the West (71.43%), gaining spiritual enlightenment on truth, goodness, and beauty (65.58%), fostering love for the Party, country, and people (61.04%), and enhancing national pride (59.09%). Additionally, 43.51% noted an increased awareness of law and discipline. The survey indicates that students are developing a respectful and inclusive attitude towards diverse cultures and values, while also cultivating a rigorous pursuit of truth and a sense of social responsibility.

# Limitations

While the study offers a detailed analysis of the integration of value education within EFL oral English textbooks at the college level, it is not without its limitations. Firstly, the sample is confined to a specific series of EFL oral English textbooks, which may restrict the generalizability of the findings. The analysis centered on the *New Inside Out* series, used by first-year students at a university in central China. As a result, these findings may not generalize to other educational levels or contexts. Additionally, focusing on a single textbook series limits the study's ability to fully capture the diversity of EFL materials or the multiple approaches to integrating morality and values into language learning. Future research could consider a broader range of textbook samples to enhance the representativeness and universality of the findings.

# Conclusion

The integration of values into EFL oral English textbooks at college level, has been an underexplored yet critical area of research. This study aimed to address this gap by examining the representation of morals and values in the widely-used EFL textbook series for English speaking. Through the lens of Language Appraisal Theory, upon an in-depth textual analysis, emerge several notable characteristics of value integration. To start with, value elements have been ingeniously integrated into various thematic and activity-based teaching modules, such as listening, speaking, reading, writing, grammar and vocabulary. These encompass a wide array of eight key values including "care and love", "love for the healthy lifestyle", "respect", "environmental awareness", "responsibility", "rule awareness", "kindness and friendliness", and "positive energy". The textbooks' emphases on these core values could be viewed as a reflection of the broader shift towards a more holistic approach to language teaching and learning, which recognizes the importance of nurturing not just academic achievement, but also moral and emotional development. This demonstrates the potential for language education to be a powerful tool for character building and value shaping. Significant differences in the distribution of values across textbook series reflect varying educational objectives and student needs. One volume emphasizes personal well-being and career development dimension, while the other

focuses on emotional and social relationship dimension. These distinct emphases suggest a tailored approach to embedding morals and values, highlighting the need for flexibility in addressing diverse educational contexts and learner profiles. By engaging with the textbook that prioritizes personal well-being and career development dimension, learners are positioned to cultivate a strong sense of self-awareness for their holistic development. Conversely, the prominence of emotional and social relationship dimension in the other textbook provides a solid framework for the development of social competencies and emotional intelligence, essential for navigating interpersonal landscape.

The integration of values within the oral English course has proven to be both popular and effective, as demonstrated by the overwhelmingly positive feedback received from learner questionnaire and their significant improvements in learners' personal growth, values, and beliefs. The prioritization of moral values by learners indicates that value-driven oral English teaching is highly regarded. This can be leveraged in oral English classes to encourage learners to articulate and defend their viewpoints on moral issues, thus practicing language skills in a meaningful context. The findings indicate that a thoughtfully designed textbook, which integrates language proficiency with moral and value considerations, can foster well-rounded learners who excel not only in language skills but also in their awareness of values.

# References

Abdul Rahim, H., & Jalalian Daghigh, A. (2019). Locally-developed vs. Global Textbooks: An Evaluation of Cultural Content in Textbooks Used in ELT in Malaysia. *Asian Englishes*, *22*(3), 317–331.

Asif, T., Guangming, O., Haider, M. A., Colomer, J., Kayani, S. & Amin, N. (2020). Moral Education for Sustainable Development: Comparison of University Teachers' Perceptions in China and Pakistan. *Sustainability, 12*(7), 3014.

Bates, A. (2019). Character education and the 'priority of recognition'. *Cambridge Journal of Education, 49*(6), 695–710.

Briggs, N., & Kim, Y. (2020). The Moral Dimension of English Language Teaching: Exploring the Collaborative Reflections of Two In-Service University Instructors. *English Teaching & Learning, 44,* 331–352.

Coleman, E., & Leider, M. (2013). Personal and Professional Growth Realized: A Self-Study of Curriculum Design and Implementation in a Secondary Science Classroom. *Studying Teacher Education*, *10*(1), 53–69.

Feng, W. D. (2017). Infusing moral education into English language teaching: an ontogenetic analysis of social values in EFL textbooks in Hong Kong. *Discourse: Studies in the Cultural Politics of Education*, *40*(4), 458–473.

Gao, F. (2020). Negotiation of native linguistic ideology and cultural identities in English learning: a cultural schema perspective. *Journal of Multilingual and Multicultural Development.* Advance online publication.

Han, H., Park, S. C., Kim, J., Jeong, C., Kunii, Y., & Kim, S. (2018). A quantitative analysis of moral exemplars presented in moral education textbooks in Korea and Japan. *Asia Pacific Journal of Education,* 38(1), 62-77.

Herrera, L. J. P. (2019). How to Behave and Why: Exploring Moral Values and Behavior in the ESOL Newcomer Classroom. *TESOL Quarterly*, *53*(4), 1033–1059.

Keskin, Y. (2013). A Comparison between Turkish and British Students' Acquisition Levels of Universal-Social Values Placed in Curriculum: A Case Study. *The Anthropologist*, *16*(1–2), 185–201.

Khan, Y. (1998). *Japanese moral education past and present.* Madison, NJ: Fairleigh Dickinson University Press.

Kusramadhani, S., Nirwanto, R., & Widiastuty, H. (2022). Moral Values in English Textbook: ''When English Rings a Bell.'' *PROJECT: Professional Journal of English Education, 5*(2), 344-353.

Li, D. (2023). Multimodal Analysis of Image Texts in College English Textbooks Using Visual Grammar Theory for Real-Time Embedded Systems. *Scalable Computing: Practice and Experience, 24*(4), 1137–1144.

Lin, J. C., & Jackson, L. (2022). Patriotism in moral education: Toward a rational approach in China. *Journal of Moral Education*, *52*(3), 343–361.

Liu, Y. (2023). Neoliberal personhood as exception: A critical analysis of textbooks of China’s moral education. *Journal of Moral Education, 38*(1), 62-77.

Lovat, T. (2017). Values education as good practice pedagogy: Evidence from Australian empirical research. *Journal of Moral Education, 46*(1), 88-96.

Lu, J., Liu, Y., An, L., & Zhang, Y. (2022). The cultural sustainability in English as foreign language textbooks: Investigating the cultural representations in English language textbooks in China for senior middle school students. *Frontiers in Psychology, 13*, 944381.

Mangubhai, F. (2007). The moral and ethical dimensions of language teaching.

*Australian Journal of Education, 51* (2), 178–189.

Martin, J.R., & White, P.R.R. (2005). *The Language of Evaluation: Appraisal in English.* Palgrave Macmillan.

Meyer, J.E. (1988). Teaching Morality in Taiwan Schools: The Message of the Textbooks. *The China Quarterly, 114,* 267–284.

Pentón Herrera, L. J. (2019). How to behave and why: Exploring moral values and behavior in the ESOL newcomer classroom. *TESOL Quarterly, 0*(0), 1-27.

Phan, L. H., & Phan, V. Q. (2006). Vietnamese Educational Morality and the Discursive Construction of English Language Teacher Identity. *Journal of Multicultural Discourses, 1*(2), 136-151.

Prahaladaiah, D. (2021). Value-based teaching English language and literature.

*Journal of Education Culture and Society, 2*, 59-66.

Pratiwi, Y., Andajani, K., Suyitno, I., Ismail, A., & Prastio, B. (2023). Representing and Implementing Moral Values to Foreign Students in Indonesian Textbooks for Learners Other Than Indonesians. *International Journal of Language Education, 7*(1), 58-76.

Qoyyimah, U. (2016). Inculcating character education through EFL teaching in Indonesian state schools. *Pedagogies: An International Journal*, *11*(2), 109–126.

Qoyyimah, U., Singh, P., Exley, B., Doherty, C., & Agustiawan, Y. (2020). Professional Identity and Imagined Student Identity of EIL Teachers in Islamic Schools. *Journal of Language, Identity & Education*, *22*(2), 121–136.

Soleimani, N., & Lovat, T. (2019). The cultural and religious underpinnings of moral teaching according to English language teachers' perceptions: a case study from Iran. *Journal of Beliefs & Values*, *40*(4), 477–489.

Rahim, H. A., & Daghigh, A. J. (2020). Locally-developed vs. Global Textbooks: An Evaluation of Cultural Content in Textbooks Used in ELT in Malaysia. *Asian Englishes, 22*(3), 317-331.

Roh, Y.-R. (2004b). *Values education in the global, information age in South Korea and Singapore*. Hong Kong, China: Kluwer Academic Publishers.

Saldivar, R., Herjanto, H., & Adiwijaya, M. (2024). Personal values matter: international students’ university selection. *Studies in Higher Education*, 1–18.

Song, B. (2019). Exploring the Cultural Content in Chinese ELT Textbooks from Intercultural Perspectives. *The Journal of Asia TEFL, 16* (1), 267-278.

Tse, T. K., & Zhang, L. (2017). Changing mode of regulation: Role models in moral education textbooks for primary schools in China, 1999–2005. *Frontiers of Education in China, 12*(1), 52–74.

Wang, D. (2016). Learning or becoming: Ideology and national identity in textbooks for international learners of Chinese. *Cogent Education, 3*(1), 1140361.

Xiong, T. (2012). Essence or practice? Conflicting cultural values in Chinese EFL textbooks: a discourse approach. *Discourse: Studies in the Cultural Politics of Education*, *33*(4), 499–516.

Ye, W. (2022). An analysis of Hong Kong Moral and National Education textbook- teacher interactions. *Pedagogy, Culture & Society*, *32*(3), 815–833.

Zhang, X., & Lütge, C. (2023). Home culture and its effects on English as a lingua franca communication: Voices from Chinese students at a United Kingdom university. *Frontiers in Psychology,14,* 1057315.

国家教材委员会[The National Textbook Committee].(2021, September 29). 国家教材委员会关于印发《*“*党的领导*”*相关内容进大中小学课程教材指南》的通知

*[The Guidelines for Incorporating Content Related to 'Party Leadership' into Curricula for Primary, Secondary and Tertiary Schools].* Retrieved May 18, 2023, from <http://www.moe.gov.cn/srcsite/A26/s8001/202110/t20211015_572633.html>

教育部高等学校大学外语教学指导委员会[The College Foreign Language

Teaching Guidance Committee of the Ministry of Education]. (2020). 大学英语教学指南（*2020* 版）*[The Guide for College English Teaching (2020 ed.)].* 高等教育出版社. [Higher Education Press].
