# Supplementary material for: Moral integration influences English as a Foreign Language (EFL) oral English learning: Evidence from textbook analysis and learner feedback
Source: PLoS One. 2026 Feb 6;21(2):e0340479. doi: 10.1371/journal.pone.0340479 (PMC12880721; doi:10.1371/journal.pone.0340479)
Supplement: Attachment — Submitted filename: Response to Reviewers and Editors20251219.docx [file pone.0340479.s007.docx]

**Response to Editors and Reviewers**

First and foremost, I would like to express my sincere gratitude to the academic editor and anonymous reviewers for their meticulous review of this manuscript and valuable revision suggestions. Their comments from various perspectives have provided important insights for improving the paper and played a crucial role in enhancing the research quality. Below are my detailed responses to each comment, along with the corresponding revisions made to the manuscript. The comments are addressed in the order of Academic Editor, Reviewer 1, Reviewer 2, and Reviewer 3.

**To Academic Editor.**

Journal requirements:

When submitting your revision, we need you to address these additional requirements.

**Comment 1:**

1. Please ensure that your manuscript meets PLOS ONE's style requirements, including those for file naming. The PLOS ONE style templates can be found at

<https://journals.plos.org/plosone/s/file?id=wjVg/PLOSOne_formatting_sample_main_body.pdf>

and

<https://journals.plos.org/plosone/s/file?id=ba62/PLOSOne_formatting_sample_title_authors_affiliations.pdf>

**Response 1:**

Thank you very much for providing the PLOS ONE style templates. We have carefully revised the manuscript in strict accordance with the specified requirements, including adjustments to the title format, line spacing, titles of tables and figures, in-text citations, reference list, and image formats. All formatting elements now comply with the journal’s style guidelines.

**Comment 2:**

2. Thank you for stating the following financial disclosure:

[This paper is supported by the Educational Reform Research Project of Jiangxi Agricultural University, with the project title ''Deep Reform and Practice of College English Speaking under the Background of Great Ideological and Political Education'', grant number 2023B2ZZ53.].

Please state what role the funders took in the study. If the funders had no role, please state: "The funders had no role in study design, data collection and analysis, decision to publish, or preparation of the manuscript."

If this statement is not correct you must amend it as needed.

Please include this amended Role of Funder statement in your cover letter; we will change the online submission form on your behalf.

**Response 2:**

OK, thanks. We would like to add this statement into revision “**The funders had no role in study design, data collection and analysis, decision to publish, or preparation of the manuscript.**”

**Comment 3:**

3. We note that your Data Availability Statement is currently as follows: [**All relevant data are within the manuscript and its Supporting Information files.**]

Please confirm at this time whether or not your submission contains all raw data required to replicate the results of your study. Authors must share the “minimal data set” for their submission. PLOS defines the minimal data set to consist of the data required to replicate all study findings reported in the article, as well as related metadata and methods (https://journals.plos.org/plosone/s/data-availability#loc-minimal-data-set-definition).

For example, authors should submit the following data:

- The values behind the means, standard deviations and other measures reported;

- The values used to build graphs;

- The points extracted from images for analysis.

Authors do not need to submit their entire data set if only a portion of the data was used in the reported study.

If your submission does not contain these data, please either upload them as Supporting Information files or deposit them to a stable, public repository and provide us with the relevant URLs, DOIs, or accession numbers. For a list of recommended repositories, please see https://journals.plos.org/plosone/s/recommended-repositories.

If there are ethical or legal restrictions on sharing a de-identified data set, please explain them in detail (e.g., data contain potentially sensitive information, data are owned by a third-party organization, etc.) and who has imposed them (e.g., an ethics committee). Please also provide contact information for a data access committee, ethics committee, or other institutional body to which data requests may be sent. If data are owned by a third party, please indicate how others may request data access.

**Response 3:**

Thank you for reminding. In resubmission, as required we upload all the supporting files alongside the revised manuscript, including verbal texts from the studied textbooks, moral elements frequency, moral dimension comparison, questionnaire raw data.

**Comment 4:**

4. When completing the data availability statement of the submission form, you indicated that you will make your data available on acceptance. We strongly recommend all authors decide on a data sharing plan before acceptance, as the process can be lengthy and hold up publication timelines. Please note that, though access restrictions are acceptable now, your entire data will need to be made freely accessible if your manuscript is accepted for publication. This policy applies to all data except where public deposition would breach compliance with the protocol approved by your research ethics board. If you are unable to adhere to our open data policy, please kindly revise your statement to explain your reasoning and we will seek the editor's input on an exemption. Please be assured that, once you have provided your new statement, the assessment of your exemption will not hold up the peer review process.

**Response 4:**

Yes, we follow the data sharing rule. In order to reduce misunderstanding, we would like to change the data availability statement from “make data available on acceptance” to “**All relevant data are within the manuscript and its Supporting Information files**”, since all supporting data are submitted alongside the revised manuscript.

**Comment 5:**

4. If the reviewer comments include a recommendation to cite specific previously published works, please review and evaluate these publications to determine whether they are relevant and should be cited. There is no requirement to cite these works unless the editor has indicated otherwise.

**Response 5:**

Yes, upon verification, the reviewer’s recommended reference is highly related and useful, so it is added in the revision. For details, please see To Reviewer 3 Comment 11.

**Comment 6:**

5. Please review your reference list to ensure that it is complete and correct. If you have cited papers that have been retracted, please include the rationale for doing so in the manuscript text, or remove these references and replace them with relevant current references. Any changes to the reference list should be mentioned in the rebuttal letter that accompanies your revised manuscript. If you need to cite a retracted article, indicate the article’s retracted status in the References list and also include a citation and full reference for the retraction notice.

**Response 6:**

There’s no referenced article retracted, but there are indeed some changes in the reference. For detailed changes, please see: To Reviewer 1 Comment 1-9,11,12; To Reviewer 3 Comment 3,4 7,11.

**To Reviewer 1.**

**Comment 1:**

A. In the References section:

1. Abdul Rahim, H., & Jalalian Daghigh, A. (2019) is not in this manuscript excerpt. It should be removed from the references section

**Response 1:**

Agreed. Upon verification, this reference was duplicated and contained formatting errors in the author name and publication year. It has been removed from the revised manuscript.

**Comment 2:**

A. In the References section:

2. The writing of the Volume of the articles are not italicized, Check Afif et al (2020), Bates (2019), Briggs & Kim (2020), Coleman & Leider (2013), Feng (2017), Herrera (2019), Keskin (2013), Kusramadhani et al., (2022), Li (2023), and Lin & Jackson (2022), and the rest. Almost all volumes of the article in the references are written in italics. It should be changed to upright.

**Response 2:**

Agreed. All italicized volume numbers in the reference list have been changed to upright.

**Comment 3:**

A. In the References section:

3. Pratiwi (2023) in the citation should be Pratiwi et al (2023) because the author is not 1 person

**Response 3:**

Agreed. As this reference has multiple authors, it has been revised to (Pratiwi et al., 2023) in the citation.

Location in the revised manuscript (marked-up copy): Page 3, Line 111√

**Comment 4:**

A. In the References section:

4. Lin, J. C., & Jackson, L. (2022) in the citation in 2023, while in the references it is written in 2022. Need to check.

**Response 4:**

Agreed. Upon verification, the citation information for the reference—Lin, J. C., & Jackson, L. (2023). Patriotism in moral education: Toward a rational approach in China. Journal of Moral Education, 52(3), 343–361—confirms that the publication year is 2023. Thus, the citation year in the main text is correct, while the year listed in the reference list was incorrect. It has been corrected to 2023 in the reference list.

Location in the revised manuscript (marked-up copy): Page 45, Line1404√

**Comment 5:**

A. In the References section:

5. In the references it is written Qoyyimah, U., Singh, P., Exley, B., Doherty, C., & Agustiawan, Y. (2020) in the citation written Qoyyimah et al., (2023). Need to check what year.

**Response 5:**

Agreed. Upon verification, the citation information for the reference—Qoyyimah, U., Singh, P., Exley, B., Doherty, C., & Agustiawan, Y. (2023). Professional Identity and Imagined Student Identity of EIL Teachers in Islamic Schools. Journal of Language, Identity & Education, 22(2), 121–136—confirms that the publication year is 2023. Thus, the citation year in the main text is correct, while the year listed in the reference list (2020) was incorrect. It has been corrected to 2023 in the reference list.

Location in the revised manuscript (marked-up copy): Page 49, Line 1498√

**Comment 6:**

A. In the References section:

6. In the references it is written Pentón Herrera, L. J. (2019). It is better to start from the surname to Herrera, P. l. J. (2019).

**Response 6:**

Agreed. Upon verification, we change this reference to follow the requested format as below:

Herrera LJP. How to Behave and Why: Exploring Moral Values and Behavior in the ESOL Newcomer Classroom. TESOL Quarterly. 2019;53(4): 1033–1059.

Location in the revised manuscript (marked-up copy): Page 47, Lines 1436-1437√

Furthermore, we have corrected the incorrect in-text citation format and removed the given name “Pentón”.

Location in the revised manuscript: Page 7, Line 208; Page 11, Line 320√

**Comment 7:**

A. In the References section:

7. The Guide for College English Teaching (2020 edition should be included in the references

**Response 7:**

Agreed. Upon verification, this reference was already included as the last entry in the original reference list. However, it may not have been identified by the reviewer due to non-standard formatting. Therefore, we referred to the citation guidelines for non-English literature in the official reference instructions of PLOS ONE, and consulted the format of non-English article citations from the National Library of Medicine (<https://www.nlm.nih.gov/bsd/uniform_requirements.html>):

We revised the citation of this reference to: College Foreign Language Teaching Guidance Committee of the Ministry of Education. [The Guide for College English Teaching]. Beijing: Higher Education Press; 2020.

Location in the revised manuscript: Page 45 Lines 1411-1413√

Similarly, we referred to two PLOS ONE reference format samples for online sources (<https://journals.plos.org/plosone/s/submission-guidelines#loc-references>):

Published media (print or online newspapers and magazine articles): Fountain H. For Already Vulnerable Penguins, Study Finds Climate Change Is Another Danger. The New York Times. 2014 Jan 29 [Cited 2014 March 17]. Available from: <http://www.nytimes.com/2014/01/30/science/earth/climate-change-taking-toll-on-penguins-study-finds.html>

New media (blogs, web sites, or other written works): Allen L. Announcing PLOS Blogs. 2010 Sep 1 [cited 17 March 2014]. In: PLOS Blogs [Internet]. San Francisco: PLOS 2006 -. [about 2 screens]. Available from: <http://blogs.plos.org/plos/2010/09/announcing-plos-blogs/>.

Correspondingly, we revised the second-to-last entry in the original reference list: “国家教材委员会[The National Textbook Committee].(2021, September 29). 国家教材委员会关于印发《“党的领导”相关内容进大中小学课程教材指南》的通知[The Guidelines for Incorporating Content Related to 'Party Leadership' into Curricula for Primary, Secondary and Tertiary Schools]. Retrieved May 18, 2023, from <http://www.moe.gov.cn/srcsite/A26/s8001/202110/t20211015_572633.html>”

The revised format is as follows:

Ministry of Education. [Notice of the Ministry of Education on Issuing "Guidelines for Incorporating Content Related to 'Party Leadership' into Curricula for Primary, Secondary and Tertiary Schools" (Guojiaocai〔2021〕No. 5)]. 2021 September 29 [cited 2023 May 18]. In: Ministry of Education of the People's Republic of China Public Access [Internet]. Available from:

<http://www.moe.gov.cn/srcsite/A26/s8001/202110/t20211015_572633.html>

Location in the revised manuscript: Page 45, Lines 1405-1410√

**Comment 8:**

A. In the References section:

8. In the citation it is written (Lu, 2022) while in the references it is written Lu, J., Liu, Y., An, L., & Zhang, Y. (2022). In the citation it needs to be changed to (Lu et al., 2022).

**Response 8:**

Agreed. As this reference has multiple authors, it has been revised to (Lu et al., 2022) in the citation.

Location in the revised manuscript: Page 3, Line 93√

**Comment 9:**

A. In the References section:

9. Zhang, X., & Lütge, C. (2023) is not in the citation, it should be removed from the references.

**Response 9:**

Agreed. Upon verification, this reference was not cited in the text, so it has been removed from the revised manuscript.

**Comment 10:**

B. The Abstract section has introduced the research topic, the number of verbal text samples, and how to analyze the research data clearly, but has not stated the reasons for **the importance** of conducting the research, the **purpose** of conducting the research, and the **instruments for collecting data to obtain 273 verbal text samples**. Likewise, it is also necessary to explain **what the survey is like**.

**Response 10:**

Explanation of Abstract Revisions

This abstract has been revised based on the core content of the research’s Introduction, Literature Review, and Methodology sections, aiming to address all comments raised by the reviewer and enhance precision, completeness, and logical coherence. The specific revisions are as follows:

1. Clarified research motivation and purpose (replacing vague original wording)

The original opening (“The research is motivated by the importance of understanding how value education is intertwined with language learning...”) was revised to “The research is motivated by the strategic integration of moral and value education into China’s college English curriculum and the critical role of textbooks as value carriers in EFL education. Despite growing attention to value integration in EFL materials, empirical studies on moral and value representation in oral English textbooks remain sparse, and learners’ perspectives on value acquisition through oral instruction are underexplored—this study aims to address these gaps.” This revision draws on the Introduction’s emphasis on national educational policies and the pivotal role of textbooks, as well as the Literature Review’s identification of research gaps (scant focus on oral English textbooks and learner perspectives), clearly articulating the study’s academic and practical significance and core purpose (addressing existing gaps).

2. Elaborated on the method for obtaining 273 value-laden verbal text samples

The original brief description (“categorizing and analyzing verbal texts from the selected EFL oral English textbook series at the college level”) was expanded to specify details from the Methodology section: “the researcher systematically analyzed 318 pages of *New Inside Out* Volume 1 and Volume 2 (first-year college EFL oral English textbooks), screening all module types including vocabulary, grammar, dialogue, listening, reading, writing, and exercise for value-related content, which was then coded and recorded in Excel.” This clarifies the specific textbook title, scope of analysis, module coverage, and data processing steps, directly responding to the reviewer’s request for explaining the data collection instrument/method for the 273 samples.

3. Detailed the questionnaire survey design

The original vague mention (“a questionnaire survey was conducted with EFL learners who used the textbook series for an academic year”) was supplemented with key information: “to gather learner feedback on the effectiveness of value education in the oral English course, an online questionnaire survey (via Wen Juan Xing platform) was administered to learners who used the selected EFL oral English textbooks for a whole academic year.” This revision, based on the Methodology section’s survey details, specifies the survey’s purpose, carrier, and participant scope, addressing the reviewer’s requirement to explain “what the survey is like” .

4. Unified terminology and refined expressions for consistency and accuracy

Replaced “Book 1 and Book 2” with “Volume 1 and Volume 2” and adjusted “between different textbook volumes” to “between the two volumes” to ensure consistent textbook terminology;

Standardized module-related expressions: used “module types” as the general term and singular forms (dialogue, listening, reading, etc.) for specific module categories to resolve inconsistency in number usage;

Added “EFL” to the textbook description (“first-year college EFL oral English textbooks”) to align with the study’s EFL context and clarify the textbook type;

Revised “lexical-grammatical items” to “linguistic resources” (consistent with the core of Martin and White’s Appraisal Theory and the diverse module content) to avoid narrow linguistic unit references;

Condensed “verbal texts embedded with morals and values” to “value-laden verbal text samples” for academic conciseness;

Changed “values-based language instruction” to “values-based EFL instruction” to accurately reflect the study’s college EFL context and avoid overgeneralization.

All revisions are grounded in the research’s Introduction, Literature Review, and Methodology sections, addressing the reviewer’s concerns (stating research importance, purpose, sample collection method, and survey details) while enhancing the abstract’s precision, completeness, and logical coherence.

**The revised text:**

**Abstract:** The research is motivated by the strategic integration of moral and value education into China’s college English curriculum and the critical role of textbooks as value carriers in EFL education. Despite growing attention to value integration in EFL materials, empirical studies on moral and value representation in oral English textbooks remain sparse, and learners’ perspectives on value acquisition through oral instruction are underexplored—this study aims to address these gaps. The study employed a mixed-methods approach: the researcher systematically analyzed 318 pages of *New Inside Out* Volume 1 and Volume 2 (first-year college EFL oral English textbooks), screening all module types including vocabulary, grammar, dialogue, listening, reading, writing, and exercise for value-related content, which was then coded and recorded in Excel. Frequency statistics show the sample textbooks include 273 value-laden verbal text samples, with a Chi-Square Test revealing significant differences in dimension emphasis between the two volumes. Within Martin and White’s (2005) Language Appraisal Theory, qualitative textual analysis identifies the value significance of linguistic resources as a prominent feature across module types. Additionally, to gather learner feedback on the effectiveness of value education in the oral English course, an online questionnaire survey (via Wen Juan Xing platform) was administered to learners who used the selected EFL oral English textbooks for a whole academic year. The survey showed that values-based EFL instruction positively impacted morality and personal growth.

Location in the revised manuscript: Pages 1-2, Lines 11-33√

**Comment 11:**

C. In the introduction section, it is necessary to add what problems occur related to the moral content and values of the EFL English textbooks.

**Response 11:**

Agreed. Upon re-reading the introduction, we noticed that while it touches on national policy directives and the role of textbooks in fulfilling educational objectives, it fails to address the potential issues inherent to EFL textbooks—a key oversight that directly underscores the rationale for this study. To rectify this, we conducted a supplementary review of literature related to the achievement of educational goals through foreign language textbooks used in Chinese contexts. Drawing on these sources and aligning with the research questions of this paper, we have supplemented the analysis of EFL textbook-related issues.

We focused specifically on the following two studies:

徐锦芬，刘文波。大中小学外语教材思政内容一体化建设研究 [J]. 外语界，2023, (03): 16-22. [Xu, J. F., & Liu, W. B. (2023). A study on the integrated construction of ideological and political content in foreign language textbooks from primary to tertiary education. *Foreign Language World,* (3), 16-22.]

许悦婷，李展，郭强。大学英语教材使用的育人目标与效果：学生感知研究 [J]. 外语界，2024, (02): 37-44. [Xu, Y. T., Li, Z., & Guo, Q. (2024). A study on the educational goals and effects of college English textbook use: Students' perceptions. *Foreign Language World,* (2), 37-44.]

Correspondingly, these two new references have been added to the reference list.

**The revised text:**

Despite the national emphasis on imparting knowledge and nurturing character in college English teaching and textbooks’ pivotal role as value carriers, several practical questions remain to be explored regarding the imported EFL oral English materials commonly used in Chinese contexts. For one, what core moral values are embedded in these textbooks and how are they integrated into teaching modules, and can they truly align with China’s character education objectives? For another, given that “students’ development of cognition, emotions, and value systems is a phased process” (Xu, 2021), do value emphases across different volumes of the same textbook series show progressive adaptations to learners? Furthermore, can learners effectively perceive and internalize the textbook-embedded values, and how does this integration impact their language learning and personal growth? As noted, studies of student-perspective textbook use are needed to verify the educational goals of foreign language courses (Xu et al., 2024). These unresolved problems highlight the necessity of the current study.

Reference:

11.	Xu JF, Liu WB. (2023). [A study on the integrated construction of ideological and political content in foreign language textbooks from primary to tertiary education]. Foreign Language World. 2023;(3):16-22.

https://link.cnki.net/doi/10.26971/j.cnki.flw.1004-5112.2023.03.003

12.	Xu YT, Li Z, Guo Q. (2024). [A study on the educational goals and effects of college English textbook use: Students' perceptions]. Foreign Language World. 2024;(2):37-44.

https://link.cnki.net/doi/10.26971/j.cnki.flw.1004-5112.2024.02.006

Location in the revised manuscript: Page 5, Lines 143-156; Page 46, Lines 1414-1421√

**Comment 12:**

D. The methodology section of this manuscript needs to be organized systematically. First, explain specifically the 1**type of mixed method** research used and the **reasons** for applying this type. Second, explain the 2**population** and **sample or subject** of the research, as well as the **sampling technique** (Explain **the process and criteria used to select samples or participants** of the research). Third, explain the 3**tools for collecting data** and the **method/process of collecting the data.** Fourth, explain the 4**data analysis technique**. Since this research uses a Mixed Method, it is necessary to explain which data are analyzed using **quantitative** analysis and which data are analyzed **qualitatively**.

**Response 12:**

In the revision, we addressed the methodological requirements proposed by the reviewers one by one, with specific adjustments as follows:

**Sampling**

For the studied textbooks, we supplemented details including the authors, publication year, textbook level, publisher, target users, teaching scenarios, number of units, and number of themes, to provide readers with a comprehensive overview of the textbooks.

**The revised text:**

The corpus of analysis is derived from two imported textbooks, New Inside Out (Sue Kay & Vaughan Jones, 2012) — Book 1 (Pre-intermediate) and Book 2 (Intermediate) — published by Macmillan. Popular among learners worldwide, this textbook series was introduced to China by Shanghai Foreign Language Education Press. To adapt to the domestic teaching context, the press further made integrated adaptations to the series, which is now widely used in oral English classes at Chinese universities—aiming to improve the oral English proficiency of English majors and non-English majors. Each textbook contains 12 units, with one main theme and three related sub-themes per unit, and the teaching content of each unit covers seven modules: speaking, writing, reading, listening, grammar, vocabulary, and pronunciation. The teaching schedule is 8 weeks per semester, with 2 class hours per week.

Location in the revised manuscript: Pages 13-14, Lines 368-385√

**Sampling criteria:**

The reviewers explicitly requested the inclusion of sampling criteria, while the original manuscript completely lacked explanations regarding how the 27 moral elements are selected from the textbooks — a gap that could easily raise doubts. Therefore, in the revision, we referred to two guiding documents on moral education work issued by the Ministry of Education of the People’s Republic of China (which cover the primary, secondary, and tertiary education stages) and synthesized an "Overall Framework for Moral Education Content" based on these documents, using it as the basis for sampling the moral education elements. The two references are as follows:

Notice of the Ministry of Education on Issuing the "Guidelines for Moral Education Work in Primary and Secondary Schools". moe.gov.cn [Internet]. Beijing: Ministry of Education of the People’s Republic of China; c2017 [cited 2025 Nov 30]. Available from: <http://www.moe.gov.cn/srcsite/A06/s3325/201709/t20170904_313128.html>

Notice of the Ministry of Education on Issuing the "Guidelines for the Construction of Curriculum Ideology and Politics in Institutions of Higher Education".2020. gov.cn [Internet]. Beijing: Ministry of Education of the People’s Republic of China; c2020 [cited 2025 Nov 30]. Available from:

<https://www.gov.cn/zhengce/zhengceku/2020-06/06/content_5517606.htm>

**The revised text:**

The selection of moral elements in textbooks was strictly based on two national policy documents:

*Guidelines for Moral Education Work in Primary and Secondary Schools* (Ministry of Education, 2017), which outlines five broad moral education contents: Ideal and Belief Education, Socialist Core Values Education, Excellent Traditional Chinese Culture Education, Ecological Civilization Education, and Mental Health Education.

Guidelines for the Construction of Curriculum Ideology and Politics in Institutions of Higher Education (Ministry of Education, 2020), which adds Vocational Ideal and Professional Ethics Education as a key content for university students.

By cross-referencing the moral education objectives and content of these two documents (covering primary, secondary, and tertiary education stages), the researcher synthesized “the cross-stage moral education framework” (Table 1) to screen moral elements. A total of 318 pages of *New Inside Out* (Book 1 and Book 2) were read word-by-word to identify verbal texts conveying values from each module. Ultimately, 273 samples were screened from the texts of all modules in the two textbooks, containing 27 adaptive moral elements. The distribution of these moral elements within the overall moral education content framework is presented in the table below:

Table 1. The cross-stage moral education framework

| **Educational Stage** | **Moral Education Content Dimension** | **Corresponding Moral Education Elements Presented in the Textbooks** |
| --- | --- | --- |
| **Primary & Secondary (Foundation)** | Ideal and Belief Education | confidence, pride |
|  | Socialist Core Values Education | discipline, fairness, honesty and trust, kindness and friendliness, respect, responsibility, rule awareness, social awareness |
|  | Excellent Traditional Chinese Culture Education | caring and love, cultural awareness, empathy, integrity, tolerance |
|  | Ecological Civilization Education | admiration for nature, care for animals, environmental awareness, thrift |
|  | Mental Health Education | independence, love for the healthy lifestyle, positive energy |
| **University (Advanced)** | Vocational Ideal and Professional Ethics Education | career ideal, creativity, hard work, professionalism, pursuit of excellence |

The original texts of the 273 samples were extracted and recorded one by one in an Excel spreadsheet. Each sample included the following information: textbook volume (Book 1/Book 2), page number, unit, module, text content, and contained moral education elements. Through this process, the original corpus of moral education elements was established.

Location in the revised manuscript: Pages 14-16, Lines 386-435√

**Population**

The population information in the original manuscript was slightly adjusted to include the gender ratio.

**The revised text:**

The survey participants were 140 first-year non-English major students from the university, with a gender ratio of male to female being 1:0.78. They used New Inside Out (Book 1 and Book 2) to learn oral English in the two semesters of their first academic year.

Location in the revised manuscript: Page 16, Lines 437-440√

**Data Collection**

In the revised manuscript, we supplemented the method for organizing the textbook corpus. Additionally, we streamlined the description of the questionnaire survey process, retaining only the introduction of the most key and essential steps.

**The revised text:**

The research data consists of two parts. First, the primary part is the original textbook corpus used for text analysis: the 273 selected corpus samples were classified according to the moral education elements they contain (i.e., samples with the same moral education element were grouped together). Then, we calculated the frequency and percentage of each moral education element in each textbook volume, followed by their total frequency and total percentage across the two volumes. The recording, classification, and basic calculations of the original corpus were all completed in Excel.

The second part of the data is the learners' feedback, collected through a questionnaire survey. The questionnaire was developed via the online platform Wen Juan Xing (WJX) to collect freshmen’s feedback on the effectiveness of value education in the oral English course (with one academic year of textbook use). The questionnaire comprises three parts: basic information of participants (Items 1-3, e.g., grade, gender), Likert scale questions (Items 9-12, assessing attitudes towards value integration), and multiple-choice questions (Items 4-8, focusing on the implementation and impact of value-based instruction).

Prior to participation, electronic informed consent was obtained from all respondents, who were informed of the survey’s purpose, procedures, data usage, and their right to voluntary withdrawal without negative consequences. The anonymous survey (with de-identified data to protect privacy) was conducted from April 18 to May 31, 2023, with weekly reminders to improve response rates.

Of the initially distributed 140 questionnaires, 154 responses were collected (surplus due to shared IP multiple submissions). After excluding 3 non-submissions and 16 invalid responses (completion time < 35 seconds), 135 valid questionnaires were retained, yielding an effective response rate of 96.43%.

Location in the revised manuscript: Pages 16-17, Lines 443-502√

**Data analysis technique:**

The reviewers explicitly requested the paper specify which data use quantitative analysis and which use qualitative analysis. In the revised manuscript, we further clarified the approach for each research question (RQ): RQ1 is qualitative, while RQ2 and RQ3 are quantitative.

Additionally, when rechecking the chi-square test section (RQ2), we found the aggregation of moral education dimensions was insufficiently rigorous. The original text stated, "Therefore, the 27 value types were reorganized into five broader dimensions based on their conceptual correlations (as categorized in Table 1), but no basis was provided for “conceptual correlations”, making this process overly subjective. In fact, dimension aggregation should refer to the revised manuscript’s “the cross-stage moral education framework”—a framework directly based on China’s national moral education guidelines. Therefore, we made the following revisions.

**The revised text:**

To address RQ 1, we coded the original textual corpus with reference to Martin & White’s (2005) Language Appraisal Theory. The coding was conducted across three major domains—affect, judgement, and appreciation—as well as their respective subcategories. This constitutes a qualitative analysis, through which we identified the types of emotional responses, assessments of character and behavior, and evaluations of objects and performances conveyed in the samples.

To address RQ 2, two rounds of Chi-Square Tests were conducted to examine differences in moral value distribution between the two textbooks. First, frequency data of the 27 value elements were input into SPSS 26 for the initial test. However, the test failed due to dispersed frequencies across the 27 elements—68% of cells had low expected counts, resulting in insufficient statistical power.

Thus, the 27 value types were reorganized into 6 broader dimensions based on “the cross-stage moral education framework” (see Table 1). After aggregating frequencies within each dimension, the second Chi-Square Test was performed, yielding significant results: test statistics (Pearson Chi-Square, Likelihood Ratio, Fisher's Exact Test) all confirmed significant differences in moral dimension distribution between the two textbooks (p < .05), indicating distinct value emphases in Book 1 and Book 2.

To answer RQ3, the 135 valid questionnaire data were analyzed via basic quantitative descriptive analysis. For Likert scale items (measuring perceived value integration effectiveness), a 4-point scale (1 = lowest, 4 = highest) was used, with predefined levels (high: 3.5-4.0; above medium: 2.5-3.4; below medium: 1.5-2.4; low: 1.0-1.4). Average scores (M) were calculated for each item, then matched to levels to summarize overall perception. For items measuring moral teaching content perception, preferred methods, and observed changes, frequency and percentage of each option were counted to identify dominant perceptions and attitudes.

In summary, this study used mixed methods for three RQs: qualitative analysis for RQ1 (text coding/classification), and quantitative methods for RQ2 (Chi-Square Test) and RQ3 (basic descriptive analysis). This “qualitative identification - quantitative verification” design ensured conclusion comprehensiveness and reliability.

Location in the revised manuscript: Page 18-19, Lines 556-586√

**Comment 13:**

E. The results of the study are explained in this manuscript comprehensively, this is very good. It just needs to be added with the **interpretation** or discussion. A good manuscript is not enough to just explain the findings, it needs to be added **why** the findings are like that.

**Response 13:**

Agreed. We reviewed the "Results and analysis" section:

In the original manuscript, the third subsection (*Value Threads in EFL Textbook Narratives*), which conducts qualitative analysis of the textbook corpus, already includes interpretations of the moral values conveyed in the texts and aligns with the literature review. Thus, no revisions are needed for this part.

However, the first subsection (*Key Value Themes in Oral English Textbook Series*) in the original manuscript had two limitations. First, it only presented data without providing corresponding interpretations. Second, when defining major moral values, it used a 5% proportional threshold but failed to justify why 5%—rather than other percentages—was selected, which introduced a degree of subjectivity.

To address these issues, we reclassified the data using the quartile method in the revised version. This method was chosen primarily for its objectivity and alignment with data distribution: it categorizes values based on quantiles derived directly from the 27 moral elements’ proportional data (e.g., Q3 = 5.49% as the high-frequency threshold), rather than relying on arbitrarily set percentages. Although the results are slightly consistent with the original (identifying 7 primary values vs. the original 8), the quartile method eliminates the subjective threshold bias caused by arbitrarily setting a 5% cutoff and enhances the reproducibility of our classification—making the results more statistically rigorous. Correspondingly, in the subsequent textual analysis, we removed the analysis of the 8th moral element from the original manuscript, while retaining the textual analysis of the first 7 elements, which fall into the high-frequency level:

“8) Positive energy

Text 30: Reading & Vocabulary. Near a beach, there is a large stone with the following proverb engraved on it: "At 70 you are still a child; at 80 you are just a youth; and at 90, if the ancestors invite you into heaven, ask them to wait until you are 100, and then you might consider it. " (Book 1 Unit 10 p88)

Text 31: Grammar. I wish I had been less anxious. (Book 2 Unit 11 p99)

Text 30 (affect-un/happiness) conveys a positive perspective on aging, suggesting a sense of joy and optimism about life at advanced ages. The proverb engraved on the stone promotes a view of aging that is not typically associated with negative emotions, but rather with a continued sense of youthfulness and potential. Similarly, the positive perspective on aging in text 30 reflects that character education promotes psychological well-being (Bates, 2019). This perspective supports the broader educational goal of fostering positive energy and self-confidence among students, as emphasized in various educational initiatives aimed at personal development and well-being (Feng, 2017; Pratiwi et al., 2023). The expression of a wish to have been less anxious in text 31 (affect-in/security) implies a reflection on past emotions that may have detracted from the speaker's overall sense of well-being. While the statement acknowledges a feeling of insecurity, it also carries a positive energy by indicating a desire for a more relaxed approach to life's challenges. It also highlights the value of self-reflection and personal growth, which contribute to a more fulfilling and meaningful life (Asif et al., 2020). This reflection aligns with educational programs that emphasize self-regulation and social-emotional skills, aiming to enhance students' overall well-being and self-motivation (Han et al., 2018; Mangubhai, 2007).” (Removed)

Furthermore, in the Data Collection section, the original manuscript had an insufficiently rigorous method for aggregating moral education dimensions when addressing RQ2. To address this, we re-aggregated the dimensions in accordance with the “the cross-stage moral education framework”. This re-aggregation led to changes in variables, so we conducted the Chi-Square Test again and generated a new cross-tabulation. The results still confirmed a significant difference in the distribution of moral education content between the two textbooks.

Therefore, we used a more precise statistical method to rewrite two subsections: the first subsection (*Key Value Themes in Oral English Textbook Series*) and the second subsection (*Textbook Volume Disparity in Value Representation*).

Furthermore, in line with the reviewer’s comments, we added interpretations of the significance of the data results and the underlying reasons for these results to address the lack of detailed explanation in the original manuscript.

**The revised text:**

**Moral contents in oral English textbook series**

To objectively categorize the 27 moral value elements based on their proportional distribution, we adopted the quartile method. First, the proportions were sorted in ascending order (a statistical requirement for quartile calculation), and three key quantiles were calculated: Q1 (25th percentile) = 1.47%, Q2 (median, 50th percentile) = 2.56%, and Q3 (75th percentile) = 5.49%. Based on these quantiles, the elements were divided into three tiers from high to low frequencies (see Fig. 1 for detailed proportions and frequencies).

Fig. 1. Moral elements distribution in the textbook series.

There are 7 moral elements that fall under high-frequency values (≥5.49%): caring and love, love for the healthy lifestyle, respect, environmental awareness, responsibility, rule awareness, kindness and friendliness. Collectively accounting for ~56.8% of total occurrences, these values reflect the textbooks' core focus on three dimensions of the moral education framework: "Excellent Traditional Chinese Culture Education", "Socialist Core Values Education", and "Ecological Civilization Education".

The 7 high-frequency moral elements align with regional and East Asian EFL textbook moral trends, supported by existing literature. First, environmental awareness' high frequency reflects contemporary moral priorities, consistent with Tse & Zhang (2017), who noted newer Chinese primary moral textbooks emphasize protecting the environment—validating this value's presence here and extending their findings to higher education. Next, love for the healthy lifestyle and caring and love co-occur in this tier, mirroring Feng (2017) 's Hong Kong EFL textbook findings that East Asian EFL values progress from personal (e.g., healthy lifestyle) to interpersonal domains. Notably, respect gains cross-regional support from Han et al. (2018). Their Korean-Japanese moral textbook comparison highlighted respect for human life as a shared East Asian core value, reinforcing that respect's high frequency here aligns with broader regional educational consensus.

11 moderate-frequency values (1.47% < proportion < 5.49%) include positive energy, social awareness, creativity, pride, empathy, honesty and trust, career ideal, cultural awareness, thrift, admiration for nature, fairness. Contributing ~35.2% to the total, this tier supplements the core values with diverse orientations and covers all moral education dimensions for primary and secondary education, with moderate representation of university-level values (career ideal, creativity).

The 11 moderate-frequency elements serve as supplementary moral content, align with regional EFL textbook moral design principles, supported by the following studies. Notably, social awareness' moderate frequency mirrors two Indonesian findings: Kusramadhani et al. (2022) identified it as a prominent value in Indonesian junior high English textbooks (conveyed via implicit dialogues), while Pratiwi et al. (2023) noted Indonesian textbooks emphasize 13 social harmony-focused themes for foreign learners. This validates its presence here, reflecting cross-regional consensus on such supplementary values. For empathy, its inclusion echoes Bates (2019), who argued character education should prioritize recognition—empathetic connection to others. Though Bates focused on English schools, her emphasis on relational skills supports why empathy supplements high-frequency respect, consistent with East Asian EFL textbooks' interpersonal focus (Feng, 2017). Cultural awareness' moderate presence gains support from two regional studies: Rahim & Daghigh (2020) found Malaysian ELT textbooks prioritize intercultural content for learners' skill needs, while Pratiwi et al. (2023) noted Indonesian textbooks integrate cultural values with accompanying moral dimensions into communication exercises. This confirms its supplementary role, aligning with global EFL design that balances cultural literacy and language learning.

The remaining 9 moral elements belong to low-frequency values (≤1.47%): hard work, independence, confidence, tolerance, care for animals, discipline, pursuit of excellence, integrity, professionalism. With a total proportion of only ~8.0%, most elements belong to the framework's university-level "Vocational Ideal and Professional Ethics Education" dimension.

The low frequency of vocational values (e.g., professionalism, pursuit of excellence) is consistent with the textbook's freshman oral English positioning: first-year college students have minimal professional exploration needs, ensuring priority is given to language competence improvement. Next, as Tse & Zhang (2017) noted in their study, Chinese primary moral textbooks highlight hardworking attitude and loving study as core values, the presence of hard work in the current textbook further confirms that this value is an outstanding quality consistently emphasized across primary, secondary, and university education stages.

**Textbook volume disparity in value representation**

Based on a total of 273 samples (Table 2), a Chi-Square Test was conducted to examine the distribution differences of moral education dimensions between Book 1 and Book 2. The results of the overall test showed that the distribution of moral education dimensions differed significantly across the two volumes (Pearson χ² = 39.593, df = 5, *p* < 0.001; Likelihood Ratio χ² = 41.493, df = 5, *p* < 0.001; Fisher's Exact Test = 40.439, *p* < 0.001) (Table 3).

Table 2. Case Processing Summary.

| Moral Education Dimensions * Volumes | Valid | | Missing | | Total | |
| --- | --- | --- | --- | --- | --- | --- |
|  | N | Percent | N | Percent | N | Percent |
|  | 273 | 100.0% | 0 | 0.0% | 273 | 100.0% |

Table 3. Chi-square test.

|  | Value | df | Asymptotic Significance (2-sided) | Exact Sig. (2-sided) |
| --- | --- | --- | --- | --- |
| Pearson Chi-Square | 39.593^a^ | 5 | .000 | .000 |
| Likelihood Ratio | 41.493 | 5 | .000 | .000 |
| Fisher's Exact Test | 40.439 |  |  | .000 |
| N of Valid Cases | 273 |  |  |  |

a. 0 cells (0.0%) have expected count less than 5. The minimum expected count is 6.36.

Post hoc comparisons, derived from the Moral education dimension * volumes crosstabulation (Table 4), were based on subscript letters a and b—where different letters indicate significant differences between Book 1 and Book 2 at the 0.05 level, and the same letter indicates no significant variation. These comparisons showed that four dimensions exhibited significant between-book differences (marked with ** in Fig 2): Ecological Civilization Education (Book 1: 22[a], Book 2: 12[b]) and Mental Health Education (Book 1: 35[a], Book 2: 15[b]) were more prominent in Book 1, whereas Excellent Traditional Chinese Culture Education (Book 1: 11[a], Book 2: 44[b]) and Vocational Ideal and Professional Ethics Education (Book 1: 5[a], Book 2: 19[b]) were emphasized more in Book 2. No statistically significant differences were found in Ideal and Belief Education (Book 1: 9[a], Book 2: 5[a]) and Socialist Core Values Education (Book 1: 42[a], Book 2: 54[a]). All expected frequencies exceeded 5 (minimum expected count = 6.36) (Table 3), meeting the prerequisite for the Chi-Square Test, and the consistency of multiple statistical indicators confirmed the reliability of the results.

Table 4. Moral education dimensions * volumes crosstabulation.

|  | | | Volumes | | Total |
| --- | --- | --- | --- | --- | --- |
|  |  |  | Book1 | Book2 |  |
| Moral Education Dimensions | Ecological Civilization Education | Count | 22_a_ | 12_b_ | 34 |
|  |  | Expected Count | 15.4 | 18.6 | 34 |
|  | Excellent Traditional Chinese Culture Education | Count | 11_a_ | 44_b_ | 55 |
|  |  | Expected Count | 25 | 30 | 55 |
|  | Ideal and Belief Education | Count | 9_a_ | 5_a_ | 14 |
|  |  | Expected Count | 6.4 | 7.6 | 14 |
|  | Mental Health Education | Count | 35_a_ | 15_b_ | 50 |
|  |  | Expected Count | 22.7 | 27.3 | 50 |
|  | Socialist Core Values Education | Count | 42_a_ | 54_a_ | 96 |
|  |  | Expected Count | 43.6 | 52.4 | 96 |
|  | Vocational Ideal and Professional Ethics Education | Count | 5_a_ | 19_b_ | 24 |
|  |  | Expected Count | 10.9 | 13.1 | 24 |
| Total | | Count | 124 | 149 | 273 |
|  |  | Expected Count | 124 | 149 | 273 |

Each subscript letter denotes a subset of Volumes categories whose column proportions do not differ significantly from each other at the .05 level.

Fig 2. Moral education dimension comparison.

Location in the revised manuscript: Pages 19-25, Lines 590-719√

**Comment 14:**

F. In the limitation section, it is necessary to **add the limited questions in the survey (only 4 questions) to ask learner attitudes and preferences on value integration is not comprehensive**.

**Response 14:**

Agreed. We add “Thirdly, only four questions are used to investigate learner perception of value integration, which is not comprehensive enough” to the limitation part.

Location in the revised manuscript: Page 42, Lines 1253-1254√

**Comment 15:**
G. At the end of this manuscript, the implications of this study need to be added.

**Response 15:**

Agreed. There was indeed no implication provided in the original manuscript; therefore, during the revision, we have put forward targeted suggestions based on the results of each RQ right before the “Limitations” section, which are supplemented as follows:

**The revised text:**

**Implications**

Textbook writers should optimize freshman-oriented textbooks by retaining high-frequency foundational moral elements as core moral content, properly allocating moderate-frequency supplementary elements to enrich diversity, and reducing low-frequency elements, ensuring the element distribution aligns with the “the cross-stage moral education framework” (Table 1).

Teachers should first identify frequent moral elements within the textbook modules, then play a role in shaping values by involving students in moral-embedded language knowledge building and language skill acquisition. This helps students develop sound worldviews, outlooks on life, and right values (Ministry of Education, 2020) while improving language abilities. Teachers should also pay attention to the significant differences in moral education distribution between Book 1 and Book 2 of *New Inside Out*. Specifically, when teaching Book 1, focus on its strengths in Ecological Civilization Education and Mental Health Education to design relevant oral activities; when teaching Book 2, emphasize its focus on Excellent Traditional Chinese Culture Education and Vocational Ideal and Professional Ethics Education for targeted practice. As it is noted by Xu (2023) that textbook objectives should be designed based on students’ cognitive levels at corresponding stages and reflect differences in hierarchy and depth across stages.

Based on students’ preferred choices of teaching methods in value-integrated oral English classes, teachers could center around methods such as video watching, topic discussion, role-playing, thematic speeches. Allow learners to have a deeper understanding of morals through interactive and multimodal situations.

Location in the revised manuscript: Pages 40-41, Lines 1219-1241√

**The author’s new change in Conclusion:**

Due to the revisions in the main part of the paper (including high-frequency level elements, moral dimensions, suggestions for textbook writers and teachers)，we make necessary changes to the **conclusion** part.

**The revised text:**

The integration of moral values into college-level EFL oral English textbooks is an underexplored yet critical area, as language education increasingly serves as a vehicle for individual holistic development. This study addressed this research gap by examining the representation of moral values in a widely used EFL oral English textbook series, drawing on the Language Appraisal Theory to conduct in-depth textual analysis and statistical validation.

Several key findings emerged from the study. Firstly, moral values are ingeniously embedded in thematic and activity-based language skill modules. This emphasis reflects the shift toward holistic language teaching—one that prioritizes not only linguistic proficiency but also moral and emotional development. These values focus on seven high-frequency core values: "care and love", "love for the healthy lifestyle", "respect", "environmental awareness", "responsibility", "rule awareness", and "kindness and friendliness".

Secondly, the distribution of moral values differs significantly between the two textbook volumes, conforming to the developmental characteristics of the academic stage. Specifically, four moral education dimensions exhibit significant differences across the two volumes: Ecological Civilization Education, Mental Health Education, Excellent Traditional Chinese Culture Education, and Vocational Ideal and Professional Ethics Education. In contrast, Ideal and Belief Education and Socialist Core Values Education show no statistically significant differences between the two volumes, reflecting their role as foundational values consistently emphasized across both.

What's more, the value-integrated instruction proves effective—learner questionnaire survey reveals noticeable positive feedback, with improvements in learners' personal growth, value awareness, and interpersonal skills.

Based on the study we therefore suggest that textbook writers follow the "Overall Framework for Moral Education Content" to optimize freshmen-oriented textbooks by retaining high-frequency moral elements as core values, supplementing moderate-frequency elements to enrich diversity, and reducing low-frequency elements. For teachers, adopting students' preferred methods (e.g., video watching, topic discussion, role-playing, thematic speeches) in value-integrated oral English classes is advisable, as these interactive methods help nurture learners' sense of morality and translate attitudes into tangible behaviors.

Location in the revised manuscript: Pages 42-43, Lines 1259-1293√

**To Reviewer 2:**

**Comment 1:**

1. Introduction: as the fundamental section in which the background knowledge, significant phenomena or issues, and the synthesizing theory and related previous studies, the author has to describe and explain the hypothesis or research questions at the end of the introduction.

**Response 1:**

Partially agreed. Upon careful verification, the research questions requested by the reviewer have been proposed in the Literature Review, following the identification of research gaps at the end of the section. However, a detailed re-examination revealed that Research Questions 1 and 3 in the original manuscript correspond to the identified research gaps, while Research Question 2 lacks a corresponding research gap, resulting in an oversight. To address this, we re-read the Literature Review and, drew on Keskin (2013), and supplemented the missing research gap for Research Question 2 between the two original gaps as follows:

**Second, few studies have investigated how moral and value emphases vary across different volumes of the same EFL oral English textbook series—an oversight given that “contents and target acquisition levels of values should be classified taking students’ development levels into consideration” (Keskin, 2013).**

Location in the revised manuscript: Pages 11-12, Lines 324–329√

**Comment 2:**

All the sub title do not follow the rules of title, author has correct all of them.

**Response 2:**

Agreed. We carefully revised titles and subtitles format one by one.

**Response to reviewer 3**

**Comment 1:**

Please make a brief and concise title reflecting your ideas that you are going to discuss further. In term of naming EFL, it should be written clearly what it stands for. Make your title into a declarative sentence.

**Response 1:**

Agreed. We revised the title to “Moral Integration Influences English as a Foreign Language (EFL) Oral English Learning: Evidence from Textbook Analysis and Learner Feedback”

Location in the revised manuscript: Page 1, Lines 1-3√

**Comment 2:**

Introduction of research paper should begin by broadly introducing topic, then narrowing to focus on research questions or hypothesis. Please add research question or hypothesis.

**Response 2:**

Reviewer 1 raised the same comment; therefore, the revision content here remains consistent, and I have copied and pasted it directly below. Please review. Upon careful verification, the research questions requested by the reviewer have been proposed in the Literature Review, following the identification of research gaps at the end of the section. However, a detailed re-examination revealed that Research Questions 1 and 3 in the original manuscript correspond to the identified research gaps, while Research Question 2 lacks a corresponding research gap, resulting in an oversight. To address this, we re-read the Literature Review and, drew on Keskin (2013), and supplemented the missing research gap for Research Question 2 between the two original gaps as follows:

Second, few studies have investigated how moral and value emphases vary across different volumes of the same EFL oral English textbook series—an oversight given that “contents and target acquisition levels of values should be classified taking students’ development levels into consideration” (Keskin, 2013).

Location in the revised manuscript: Pages 11-12, Lines 324–329√

**Comment 3:**

Please explain more regarding role of value education based on your point of view, by adding one example of phenomena or issue happened. Moreover, you have to put the relevant reference as a proof.

**Response 3:**

Agreed. Indeed, the original text did not elaborate on the role of value education. In our revision, we refer to documents from the Ministry of Education of China for citation and explanation, while removing redundant information from this paragraph of the original draft.

The reference is put in the reference list:

Notice of the Ministry of Education on Issuing the "Guidelines for the Construction of Curriculum Ideology and Politics in Institutions of Higher Education".2020

gov.cn [Internet]. Beijing: Ministry of Education of the People’s Republic of China; c2020 [cited 2025 Nov 30]. Available from:

<https://www.gov.cn/zhengce/zhengceku/2020-06/06/content_5517606.htm>

**The revised text:**

In the educational landscape, the indispensable role of value education in shaping well-rounded individuals is increasingly recognized. As emphasized by the Ministry of Education of China (2020), "It is an inherent requirement of talent cultivation to integrate value guidance into knowledge impartment and competence development, thereby helping students form correct worldviews, outlooks on life, and values" — this statement precisely clarifies the core role of value education: it is not isolated from teaching, but deeply integrated with knowledge and competence development, fundamentally laying a solid ideological foundation for individual growth.

Location in the revised manuscript: Pages 2-3, Lines 38-95√

**Comment 4:**

Please put reference according to APA

**Response 4:**

Agreed. In the original manuscript, we didn’t quote the source information. So we add “Hong Kong Curriculum Development Council,2004”, and put it in the reference list (following the journal’s reference format).

**The revised text:**

The strategic moral and value integration into the curriculum is further underscored in the English Language Education Key Learning Area Curriculum Guide (Hong Kong Curriculum Development Council,2004).

Reference:

3.	Hong Kong Curriculum Development Council. English language education key learning area curriculum guide (Primary 1–6). Hong Kong: Government Printer; 2004.

Location in the revised manuscript: Page 3, Line 102; Page 44, Lines 1384-1386√

**Comment 5** **(within the same paragraph):**

Please explain and support with examples or supporting ideas and add reference as proof.

It is better to reference from original text and author.

If you reference from the original text, it will be better

**Response 5:**

Due to time constraints, we are unable to locate the original text of the literature, so this paragraph is removed:

The historical narrative of moral education is deeply rooted in the evolution of state ideologies and the propagation of societal values. In Korea, the establishment of moral education as an independent subject in 1973 was a response to the nationalistic sentiments stirred by the Korean War and the ongoing tensions between the North and South, leading to the emergence of values such as patriotism and the will for unification that continue to influence the moral education curriculum (Han et al., 2018, as cited in Roh, 2004b). This mirrors the cultural approach to moral education seen in Japan with the introduction of Dotoku in 1958, emphasizing the path of virtue, and in China since 1949, where moral education has been inextricably linked with political ideologies, implementing extensive ideological education programs across all social sectors, including the schooling system (Han et al., 2018, as cited in Khan, 1998; Tse & Zhang, 2017). These historical contexts highlight how moral education has served as a tool for instilling national identity and promoting specific societal values. China's moral education curriculum contains competing ideas about the state-citizen relationship, with some values emphasizing the protection of individual freedoms and rights, while others prioritize the citizen's duty to contribute to and sacrifice for the country (Liu, 2023). (removed)

**Comment 6:**

Teacher’s role is new idea, you can discuss it separately.

**Response 6:**

Agreed. We start the following text in a new paragraph:

Teachers play a pivotal role in this process, serving as moral guides or role models, and their investment in student development encompasses both the sociopolitical and the educational, shaping their behaviors in the classroom (Briggs & Kim, 2020; Kusramadhani et al., 2022). Coleman and Leider (2013) highlight the importance of teachers positioning themselves as both educators and learners in the classroom. Their self-study demonstrates that teachers can empower themselves and their students by engaging in reflective practices and curriculum design that promote personal and professional growth. These concerns highlight the importance of value input in teaching practices (Mangubhai, 2007).

Location in the revised manuscript: Page 7, Lines 217-225√

**Comment 7:**

Please add reference for the proof of this idea.

**Response 7:**

Agreed. To supplement evidence for the idea that "a variety of educational materials and programs foster students’ environmental and social responsibility," we have added a reference to a core undergraduate English textbook for English majors in Chinese higher education: English Public Speaking: Understanding Contemporary China (a key textbook in the "Understanding Contemporary China" series) as a proof.

Reference:

Sun, Y. Z. (Chief Ed.), & Jin, L. M. (Ed.). (2022). English Public Speaking (Understanding Contemporary China Series). Beijing: Foreign Language Teaching and Research Press. ISBN 978-7-5213-3825-6.

Additionally, as this paragraph and the next one contain redundant content, we have removed the following part:

Textbooks and curricula highlight the importance of nature conservation, respect for life, and sustainable practices to cultivate a sense of responsibility and care for the environment (Han et al., 2018; Pratiwi et al., 2023). In addition, values such as respect, tolerance, and community engagement are emphasized to enhance students' understanding of social issues and their role in contributing to the greater good (Bates, 2019; Keskin, 2013; Mangubhai, 2007). These initiatives aim to develop students' appreciation for the natural world and foster a sense of compassion and responsibility towards all living beings.(removed)

**The revised text:**

Educational materials are playing a crucial role in fostering environmental and social responsibility among students. A core undergraduate English textbook for English majors in Chinese higher education—*English Public Speaking: Understanding Contemporary China* (a key textbook in the *Understanding Contemporary China Series*)—specifically dedicates independent chapters to themes of "Making Development People-Centered" and "Live Green, Live Better" [25].The former chapter integrates case studies of social responsibility, such as youth participation in poverty alleviation efforts. The latter chapter guides students to reflect on environmental problems and raise protection awareness through English speaking tasks. From such involvement, the textbook not only enhances students’ English public speaking competence but also deepens their understanding of environmental and social responsibility.

Location in the revised manuscript: Pages 8-9, Lines 241-265√

**Comment 8:**

This is not a good topic sentence of the paragraph. write topic sentence of each body paragraphs to discuss an idea about first paragraph and support with relevant supporting ideas or examples.

**Response 8:**

In fact, the following paragraph is an explanation of the three domains of the Language Appraisal Theory. Therefore, we can resolve the issue with the topic sentence by merging the next paragraph into the previous one during the revision.

Location in the revised manuscript: Pages 13, Line 350√

**Comment 9:**

Please rewrite and correct the sentence according to grammar.

**Response 9:**

Agreed. “Appreciation pertains to the evaluation of objects, phenomena, or performances and is divided into reaction (impact and quality), composition (balance and complexity), and valuation (worth).” The structure seems confusing.

**The revised text:**

Appreciation, defined as the evaluation of objects, phenomena, or performances, encompasses three key aspects: reaction (relating to impact and quality), composition (concerning balance and complexity), and valuation (pertaining to worth).

Location in the revised manuscript: Page 13, Lines 358-361√

**Comment 10:**

You are requested to use the term ‘statistically significant’ instead of ‘significant’. The omission of the term ‘statistically’ can easily result in the erroneous impression that the results are important. Hence many scholars (e.g. Carver, 1993; Wei, Liu & Wang, 2020) have underlined that the term ‘statistically’ must always precede the word ‘significant’ as dropping ‘statistically’ is one typical indicator of over-reliance on the statistical significance level (viz. p) (Wei, Yang & Wang, 2024).

References:

Carver, R. P. (1993). The case against statistical significance testing, revisited. The

Journal of Experimental Education, 61(4), 287–292.

Wei, R., Liu, H., & Wang, S. (2020). Exploring L2 grit in the Chinese EFL context.

System, 93, 102295. https://doi.org/10.1016/j.system.2020.102295

Wei, R., Yang, X., & Wang, J. (2024). Do teachers’ well-being and resilience predict

their Foreign Language Teaching Enjoyment (FLTE)? Applied Linguistics

Review, 15(6), 2515–2536. <https://doi.org/10.1515/applirev-2022-0115>

**Response 10:**

Agreed. We add “statistically” before “significantly” when reporting data where necessary.

Location in the revised manuscript: Page 2, Line 25; Page 18, Line 570; Page 43, Line 1280√

**Comment 11:**

Your manuscript situates the textbook analysis within the Chinese EFL context;

however, the scale of this setting is not immediately clear to international readers.

Providing a brief, up-to-date contextual information would help readers appreciate both the study’s scope and its broader relevance. For example, you can write something like “China had already surpassed 390 million English learners/users by 2000 (Wei & Su, 2012) and the subsequent work (e.g., Wei & Gao, 2022) suggests the continued growth”.

References:

Wei, R. & Gao, X. (2022). New research trends in TESOL: An introduction. Chinese

Journal of Applied Linguistics, 45(2), 220-223.

Wei, R. & Su, J. (2012). The statistics of English in China: An analysis of the best

available data from government sources. English Today, 28 (3), 10-14.

**Response 11:**

Agreed. We add the above sentence to the beginning of the paragraph “China had already surpassed 390 million English learners and users by 2000 (Wei & Su, 2012) [29].”

Location in the revised manuscript: Page 9, Lines 282-283; Page 48, Lines 1471-1472√

**Comment 12:**

Please use italics for statistical symbols such as p to ensure proper formatting.

**Response 12:**

Agreed. We change “p” from upright form to italic form in data reporting.

Location in the revised manuscript: Page 18, Line 572; Pages 22-23, Lines 661-687√

**Comment 13:**

I can understand that the lower and upper bounds of CI (see the screenshot of Table 4 which is reported as [.000, .000]) are very close to zero. However, to reveal a more nuanced difference, it would be commendable to keep as many digits as

possible (e.g., 99 % CI [0.0003, 0.0009]).

**Response 13:**

We sincerely appreciate your constructive suggestion on retaining more decimal places for the confidence intervals (CI) in Table 4 to reveal nuanced differences in values close to zero. The CI results reported in our study were directly generated by SPSS Statistics 26.0, with three decimal places as the software’s default output for such small values—no additional decimal digits (e.g., four decimal places) were available in the original automatic output. During revision, we have reprocessed by chi-square test as follows (SPSS does not show CI values this round):

**Table 3. Chi-square test.**

|  | **Value** | **df** | **Asymptotic Significance (2-sided)** | **Exact Sig. (2-sided)** |
| --- | --- | --- | --- | --- |
| **Pearson Chi-Square** | 39.593^a^ | 5 | .000 | .000 |
| **Likelihood Ratio** | 41.493 | 5 | .000 | .000 |
| **Fisher's Exact Test** | 40.439 |  |  | .000 |
| **N of Valid Cases** | 273 |  |  |  |

a. 0 cells (0.0%) have expected count less than 5. The minimum expected count is 6.36.

We fully understand the importance of your suggestion for data precision. If further revisions are required after the resubmission, we will try to adjust the decimal place settings in SPSS. So is this OK I’m wondering?

Location in the revised manuscript: Page 23, Lines 689-691√

**Other comments:**

All about subheading formatting such as “Apply rules of title”

Response:

Agreed. We have revised all subheadings in accordance with the PLOS ONE formatting guidelines.

<https://journals.plos.org/plosone/s/file?id=wjVg/PLOSOne_formatting_sample_main_body.pdf>

Level 1 Heading

 Use Level 1 heading for all major sections

(Abstract, Introduction, Materials and Methods, Results, Discussion, etc.).

 Bold type, 18pt font.

 Only use italics and text formatting where needed (e.g. genus and species names,

genes, etc.).

 Headings should be written in sentence case (capitalize only the first word of the

heading, the first word of the subheading, and any proper nouns and genus names)

Level 2 Heading

 Use Level 2 headings for sub-sections of major

sections.

 Bold type, 16pt font.

 Only use italics and text formatting where needed.

 Use sentence case.
